# Learning Optimal Group-structured Individualized Treatment Rules with Many Treatments

**Published:** 2023

**Authors:** Haixu Ma, Donglin Zeng, Yufeng Liu

**Affiliations:** Department of Statistics and Operations Research, University of North Carolina at Chapel Hill, Chapel Hill, NC 27599, USA; Department of Biostatistics, University of North Carolina at Chapel Hill, Chapel Hill, NC 27599, USA; Department of Statistics and Operations Research, Department of Genetics, Department of Biostatistics, Carolina Center for Genome Science, Lineberger Comprehensive Cancer Center, University of North Carolina at Chapel Hill, Chapel Hill, NC 27599, USA

**Keywords:** Angle-based multicategory classification, Group structure, Individualized treatment rules, Precision medicine, Support Vector Machine

## Abstract

Data driven individualized decision making problems have received a lot of attentions in recent years. In particular, decision makers aim to determine the optimal Individualized Treatment Rule (ITR) so that the expected specified outcome averaging over heterogeneous patient-specific characteristics is maximized. Many existing methods deal with binary or a moderate number of treatment arms and may not take potential treatment effect structure into account. However, the effectiveness of these methods may deteriorate when the number of treatment arms becomes large. In this article, we propose GRoup Outcome Weighted Learning (GROWL) to estimate the latent structure in the treatment space and the optimal group-structured ITRs through a single optimization. In particular, for estimating group-structured ITRs, we utilize the Reinforced Angle based Multicategory Support Vector Machines (RAMSVM) to learn group-based decision rules under the weighted angle based multi-class classification framework. Fisher consistency, the excess risk bound, and the convergence rate of the value function are established to provide a theoretical guarantee for GROWL. Extensive empirical results in simulation studies and real data analysis demonstrate that GROWL enjoys better performance than several other existing methods.

## 1. Introduction

A common data-driven individualized decision making problem seeks to optimize the expected value of a specified outcome, by carefully determining the Individualized Treatment Rule (ITR) based on individual characteristics and contextual information. Since the treatment effect may contain significant heterogeneity, it is necessary to tailor treatment decision rules to different subgroups of individuals. For example, using a large-scale Electronic Health Records (EHR) database, a physician may assign an optimal individualized therapy based on a patient’s specific characteristics to maximize the quality of health care (Wu et al., 2020).

Machine learning based approaches for estimating an optimal ITR have been studied intensively in the literature. These methods can be usually classified into two categories. The first category consists of model-based indirect learning methods such as modeling the conditional treatment effects given the individual characteristics (Q-learning) (Watkins, 1989; Qian and Murphy, 2011), modeling the contrast between two candidate treatment effects (A-learning) (Murphy, 2003), sub-group identification methods based on a weighted loss minimization problem (Tian et al., 2014; Chen et al., 2017), and direct learning methods (D-learning) (Qi and Liu, 2018). The second category circumvents the need for modeling conditional mean functions by directly estimating the ITR that maximizes the value function based on Inverse Probability Weighting (IPW) (Zhao et al., 2012, 2015). To combine the advantages of methods in the two categories discussed above, Zhang et al. (2012), Liu et al. (2018) and Athey and Wager (2021) proposed doubly robust augmented IPW estimation to overcome model misspecification issues. In addition, extensions to more than two treatments were studied in Zhang et al. (2021) and Qi et al. (2020).

Despite great development for estimating the optimal ITRs with a moderate number of treatments in the literature as discussed above, in some clinical problems, there can be many treatment options available. For instance, Rashid et al. (2021) analyzed the Patient-Derived Xenograft (PDX) dataset which permits the evaluation of more than 20 treatments in the allowable treatment space. Another potential challenge for learning the optimal ITR is the situation with unbalanced structure of treatment propensity scores. For example, in the Sequenced Treatment Alternatives to Relieve Depression (STAR*D) study (Rush et al., 2004), the ratio of the number of patients who were provided with the cognitive therapy and the number of patients who received venlafaxine is only around 1:3. Another example is that, when studying Type 2 Diabetes (T2D) treatment patterns, Montvida et al. (2018) concluded that the baseline treatments such as Metformin and Insulin would dominate other treatment options in the EHR database.

With many treatments but limited data size, model-based indirect methods are difficult to model conditional treatment effect due to the large number of interaction terms between treatments and features. In addition, it can be impractical to fit a useful regression model without enough observations for certain treatments. Therefore, the estimated optimal ITR induced by the indirect methods can be inaccurate with large variability due to the poor performance of the regression model. On the other hand, IPW-based direct learning methods utilize a plug-in approach for possible unbalanced propensities that appear in the denominator of IPW-based value function. Suffering from unbalanced structure of propensities in the presence of many treatments, small values in propensity scores can lead to large variability of the estimated value function.

It is interesting to point out that many treatments may work similarly for patients, due to the fact that the development of drugs is often based on intervening the same disease symptoms and mechanisms. For example, for treating depression in the STAR*D study, the 7 treatment options at Level 2 are often combined with one class of treatments involving selective serotonin reuptake inhibitors (SSRI) and the other class of treatments without SSRI because the treatments within the same class have similar treatment effects (Liu et al., 2018; Pan and Zhao, 2021). Hence, it can be helpful to identify such latent structure in the treatment space. Moreover, utilizing this latent cluster treatment structure allows us to group homogeneous treatments together and helps reducing the dimension of the treatment space. This motivates us to explore specific latent structure for treatments to identify optimal treatment groups.

To the best of our knowledge, not much has been done in the literature for estimating the optimal ITR with latent structure for treatments. Rashid et al. (2021) imposed a hierarchy binary group structure based on the conditional treatment effects for the treatments in the PDX study. This estimated group structure helps producing high-quality ITRs and identifying the important genes that are known to be associated with response to treatment. In addition, several existing methods explored combining treatment decision rules for different patients when the conditional treatment effects cannot be distinguished. Specifically, Laber et al. (2014), Ertefaie et al. (2016) and Meng et al. (2020) proposed recommending a set of near-optimal individualized treatment recommendations that are alternative to each other to a patient. However, these methods are not tailored to deal with many treatment options.

In this article, we propose estimating the latent multiple group structure of treatments and associated optimal group-structured ITRs within a single optimization. Considering grouping structure, our proposed method reduces the dimension of the treatment space and automatically clusters the treatments with similar treatment effects into the same group. In particular, we define our value function associated with both treatment partition and group-based decision rules in the IPW-based direct learning framework. The optimal treatment partition and group-based decision rules are obtained by maximizing the value function. When the treatment effects employ exact homogeneous group structure, our defined optimal partition can induce the same expected homogeneous group structure. Under the estimated optimal partition for the treatment space, the estimated group-structured ITR uses a random treatment assignment strategy, determined by randomly sampling treatment based on specific strategies within the estimated optimal treatment group. Specifically, the Reinforced Angle based Multicategory Support Vector Machines (RAMSVM) based surrogate loss function (Zhang et al., 2016) is tailored for estimating the optimal treatment group decision rules robustly in the interpretable angle-based weighted multiclass classification framework (Zhang and Liu, 2014). The group-based decision functions can give linear or non-linear decision rules to deal with complicate decision boundaries. Moreover, we prove that the surrogate loss function enjoys Fisher consistency for both group structure and group-structured ITRs. Furthermore, we present comprehensive theoretical justification on the excess risk bound, finite sample regret bound and convergence rate for our method, and allow the number of the treatment groups diverge to infinity as the sample size increases. Finally, we implement efficient algorithms to solve the non-convex integer programming problem to search for the optimal partition, and the coordinate descent algorithm to solve the dual problem of RAMSVM based weighted classification problem.

The main contributions of this article are summarized as follows. Our proposed method learns the optimal ITR by identifying the latent treatment group structure in a possible large treatment space. We cluster the treatments with similar treatment effects into the same group to reduce the dimension of the possible large treatment space. In contrast to existing methods (Zhao et al., 2012; Liu et al., 2018), our method avoids using weights involving the inverse of individual treatment propensity scores, which can be close to 0 when there are many treatments. Using the treatment group propensity scores, our method can obtain more stable estimate of the value function. In addition, our method simultaneously learns the optimal group-structured ITR and clusters the treatments. Different from the two-step method (Rashid et al., 2021), we combine both supervised learning (learn the optimal ITR) and unsupervised learning (cluster the treatments) through one single optimization. Moreover, we propose an effective procedure to determine the number of unknown treatment groups. This procedure is motivated by the trade-off between the benefit and the variability of the value function. It is worth noting that our theoretical contributions are different from that in the Outcome Weighted Learning (OWL) literature (Zhao et al., 2012). In particular, we establish the generalized Fisher consistency, excess risk bound, and finite sample regret bound with respect to both *treatment partition* and *group-based decision rule* under the angle-based multi-class classification framework.

The remainder of this article is organized as follows. In Section 2, we introduce the methodology and implementation details of our proposed GRoup Outcome Weighted Learning (GROWL) method. In Section 3, we provide theoretical guarantees of GROWL. In Section 4, we conduct simulation studies to evaluate the performance of GROWL. Our method is then illustrated using the data from the Sequenced Treatment Alternatives to Relieve Depression (STAR*D) study in Section 5. We conclude this article and discuss some future extensions in Section 6.

## 2. Methodology

In this section, we first introduce the framework of estimating optimal ITRs. Then we propose our GROWL method to estimate group-structured ITRs from the IPW-based value function.

### 2.1. Framework of Learning Optimal ITRs

Consider the i.i.d. training data $\left(X_{i},A_{i},R_{i}\right)\sim P$ for i=1,…,n, where $X_{i}\in 𝒳\subseteq R^{d}$ denotes the patient’s prognostic variables, $A_{i}\in 𝒜=\left\{1,\;2,\ldots ,M_{n}\right\}:=\left[M_{n}\right]$ is the treatment assignment, and $R_{i}\in R$ is the observed outcome for each patient i. Suppose that the number of treatments $M_{n}$ may diverge to infinity with a certain rate as the sample size n increases since we consider the large treatment space. We assume that the larger outcome is better and R is bounded. Let $(R(a){)}_{a\in 𝒜}\in R^{M_{n}}$ be the potential outcome. In addition, define the propensity function p(a∣x):=P(A=a∣X=x) and the unknown mean-outcome function μ(a∣x):=E[R(a)∣X=x] for $a\in \left[M_{n}\right]$. An Individualized Treatment Rule (ITR) D∈𝒟 is a map from the covariate space 𝒳 to the treatment space 𝒜 and $𝒟\subseteq {𝒜}^{𝒳}$ is a prespecified ITR class. Our goal is to find the optimal ITR $D^{*}\in 𝒟$, that maximizes the expected outcome, known as the value function (Zhao et al., 2012). Specifically, the value of an ITR D is defined as

$$𝒱(D)=E\left[\sum_{a=1}^{M_{n}}I[D(X)=a]R(a)\right]=E\left[\sum_{a=1}^{M_{n}}I[D(X)=a]\mu (a|X)\;\;\right].$$

Next we state the following identifiability assumptions (Rubin, 1974): (1) Consistency: $R=\sum_{a\in 𝒜}\;I[A=a]R(a)$; (2) Unconfoundedness: for each $a\in \left[M_{n}\right],R(a)⫫A|X$; (3) Positivity: p(a∣x)>0 for any x∈𝒳. If the above assumptions are satisfied, 𝒱(D) can be written as the following two equivalent forms:

(1) $$𝒱\left(D\right)=E\left[\sum_{a=1}^{M_{n}}I\left[D\left(X\right)=a]E[R|X,A=a\right]\right]$$

(2) $$\;=E\left[\frac{I\left[D\left(X\right)\;=\;A\right]}{p\left(A|X\right)}R\right].$$

Based on (1), model-based Q-learning methods (Qian and Murphy, 2011) first give an estimate for $\hat{E}[R|X,A]$ (Q-function), then the optimal ITR $D^{*}(x)$ is estimated from solving $arg\;{max}_{a\in 𝒜}\;\hat{E}[R|X=x,A=a]$. However, due to the large number of treatment options $M_{n}$ and possible unbalanced structure of the propensity score p(A∣X), we may not have enough observations for some specific treatments to fit the regression model. Consequently, $\hat{E}[R|X,A]$ can be inaccurate due to potential poor performance of the regression model and the estimated optimal ITR may have large variability. Another common approach is to estimate the value function based on (2) using empirical data, and then directly search for the optimal ITR D that maximizes the empirical value function $E_{n}\left[\frac{I[D(X)=A]}{p(A|X)}R\right]$ (Zhao et al., 2012). Note that the propensity score p(A∣X) appears in the denominator of $E_{n}\left[\frac{I[D(X)=A]}{p(A|X)}R\right]$. For the case with many treatments where insufficient data are observed for some specific treatments, it is likely to have the propensity score p(A∣X) close to 0 for some treatments. Hence, this can cause large variability of the empirical estimate for the value function.

### 2.2. GRoup Outcome Weighted Learning (GROWL)

Next we introduce our proposed GROWL method using the idea of latent group structure for the treatment space. We consider $M_{n}$ treatments can be partitioned into $K_{n}$ disjoint latent groups where $2⩽K_{n}⩽M_{n}$. We allow $K_{n}$ go to infinity with a certain rate as the sample size increases. Denote δ as the partition of 𝒜, which is a map from 𝒜 to $\left\{1,\;2,\ldots ,K_{n}\right\}:=\left[K_{n}\right]$. Under δ, denote $G_{k}^{\delta }=\{a|\delta (a)=k,a\in 𝒜\}$ as the k-th treatment set for $k\in \left[K_{n}\right]$. Intuitively, for a reasonable partition, the treatments that belong to the same treatment group should have similar treatment effects. In contrast, the treatment effects from different treatment groups should have relatively large differences. Hence, we need to first define the optimal partition $\delta^{*}$ that maximizes the expected outcome.

To start with, we define the following group-structured ITR class, denoted as $𝒟={⋃}_{\delta }\;{𝒟}_{\delta }$. Specifically, associated with a partition δ, a group-structured ITR in ${𝒟}_{\delta }$ is obtained from a random treatment assignment strategy $\pi_{\delta }$ given as

(3) $$\pi_{\delta }(a|x)=I\left[\delta (a)=D_{g}(x)\right]\frac{p(a|x)}{p(\delta (a)|x)},$$

where $D_{g}$ is a group-based decision rule mapping from 𝒳 to treatment group space $\left[K_{n}\right]$, and $p(\delta (a)|x):=P[A\in G_{\delta (a)}^{\delta }|X=x]$ is the propensity score for the δ(a)-th treatment group under δ. Then, for a given partition δ and a group-based decision rule $D_{g}$, the value function of group-structured ITR equals to the expectation of weighted conditional treatment effects. With these notations in place, we can express the value of group-structured ITR ${𝒱}_{1}\left(\delta ,D_{g}\right)$ as follows:

(4) $$\begin{array}{l} {𝒱}_{1}\left(\delta ,D_{g}\right)\;=E\left[\sum_{k=1}^{K_{n}}I\left[D_{g}\left(X\right)=k\right]\sum_{a:\delta \left(a\right)=k}\frac{p\left(a|X\right)}{p\left(\delta \left(a\right)|X\right)}E\left[R|X,A=a\right]\right]\\ \;\;=E\left[I\left[D_{g}\left(X\right)=\delta \left(A\right)\right]\frac{R}{p\left(\delta \left(A\right)|X\right)}\right]. \end{array}$$

#### Remark 1

*Based on the definition in* (3), *group-structured ITR is obtained by first estimating the group-based decision rule* $D_{g}(x)$, *then sampling one treatment from group* $G_{D_{g}(x)}^{\delta }$ *with probabilities proportional to propensity scores. Note that sampling proportional to propensity scores has the following advantages and interpretations: (a) the propensity score* p(A∣X) *showing up in the denominator of the value function in* (2) *would be cancelled by that in the sample strategy term. Thus, only the group-based propensity* p(δ(A)∣X) *appears in the value function* ${𝒱}_{1}\left(\delta ,D_{g}\right)$. *Hence, it can give a more stable estimate for* ${𝒱}_{1}\left(\delta ,D_{g}\right)$ *using empirical data especially when propensity scores are small for some treatments; (b) it makes sense to assign higher probabilities to choose prevalent treatments as they are often affordable and acceptable in practice*.

For any given δ, the optimal group-based decision rule $D_{g}^{\delta }$ is given by

(5) $$D_{g}^{\delta }\in \underset{D_{g}:𝒳\to \left[K_{n}\right]}{\arg max}{𝒱}_{1}\left(\delta ,D_{g}\right),\;$$

and the corresponding optimal value for δ is

(6) $${𝒱}_{1}^{*}\left(\delta \right)={𝒱}_{1}\left(\delta ,D_{g}^{\delta }\right).\;$$

The optimal partition $\delta^{*}$ is defined as

(7) $$\delta^{*}\in \underset{\;\delta }{\arg max}{𝒱}_{1}^{*}\left(\delta \right):=\Delta^{*},$$

where $\Delta^{*}$ is the optimal equivalent partition class and each element in this set achieves the maximum value. Observing that $\sum_{a:\delta (a)=k}\;\frac{p(a|X)}{p(\delta (a)|X)}E[R|A=a,X]=E[R|A\in G_{k}^{\delta },X]$ for $k\in \left[K_{n}\right]$, the optimal value for δ in (6) can be written as

$$\begin{array}{l} {𝒱}_{1}^{*}(\delta )\;=\underset{D_{g}:𝒳\to \left[K_{n}\right]}{max}\;E_{X}\left[\sum_{k=1}^{K_{n}}I\left[D_{g}(X)=k\right]\;E[R|A\in G_{k}^{\delta },X]\right]\\ \;=E_{X}\left[\underset{k\in \left[K_{n}\right]}{max}\;E[R|A\in G_{k}^{\delta },X]\right]. \end{array}$$

Hence, the optimal partition $\delta^{*}$ has the following interpretation. Averaging over the marginal distribution of X, the maximum of conditional treatment effects under the group domain, which is a mixture mean of conditional treatment effects under the individual treatment domain, is optimized under the optimal partition $\delta^{*}$.

It is worth noting that, when treatment effects have homogeneous structure, our defined optimal partition $\delta^{*}$ in (7) would lead to this expected natural group structure. In particular, treatment effects have homogeneous structure if treatments can be partitioned into $K_{0}$ homogeneous groups ${𝒢}^{0}=\left\{G_{1}^{0},G_{2}^{0},\ldots ,G_{K_{0}}^{0}\right\}$ and the treatment effects are identical within each treatment group set $G_{k}^{0}$ for $k\in \left[K_{0}\right]$. For each $k\in \left[K_{0}\right]$ and each pair of treatments i,j within the same treatment group $G_{k}^{0}$, we have $E[R|A=i,X]=E[R|A=j,X]=E[R|A\in \left.G_{k}^{0},X\right]$ a.e. in X. Meanwhile, for each pair of treatments r,s that belong to two different treatment groups respectively, E[R∣A=r,X]≠E[R∣A=s,X] holds with a positive probability in X. In this case, GROWL aims to combine treatments with identical treatment effects based on homogeneous structure ${𝒢}_{0}$ to reduce the dimension of treatment space and learn the optimal group-structured ITR. Denote $\delta^{0}$ to be the partition that induces the group structure ${𝒢}_{0}$, then the following Lemma 2 holds, which demonstrates that our $\delta^{*}$ is properly defined for the expected homogeneous structure.

#### Lemma 2

*Suppose* $K_{n}=K_{0}$ *and the defined optimal partition* $\delta^{*}$ *is unique. Then, we have* $\delta^{*}=\delta^{0}$.

Next we illustrate how to solve $(\delta^{*},D_{g}^{*})\in arg\;{max}_{\delta ,D_{g}:𝒳\to \left[K_{n}\right]}\;{𝒱}_{1}\left(\delta ,D_{g}\right)$ in order to get an estimate for the optimal partition $\delta^{*}$ and the associated optimal group-based decision rule $D_{g}^{*}$ under the IPW-based direct learning framework. Since

$${𝒱}_{1}\left(\delta ,D_{g}\right)+ℛ\left(\delta ,D_{g}\right)=E\left[\frac{R}{p(\delta (A)|X)}\right],$$

where the risk function $ℛ\left(\delta ,D_{g}\right):=E\left[\frac{R}{p(\delta (A)|X)}I\left[D_{g}(X)\neq \delta (A)\right]\right]$. Hence, maximizing ${𝒱}_{1}\left(\delta ,D_{g}\right)$ is equivalent to minimizing the generalized risk function:

$$\tilde{ℛ}\left(\delta ,D_{g}\right)=ℛ\left(\delta ,D_{g}\right)-E\left[\frac{R}{p\left(\delta \left(A\right)|X\right)}\right].$$

In practice, we use empirical risk minimization to approximate the generalized risk function $\tilde{ℛ}\left(\delta ,D_{g}\right)$ by $E_{n}\left[\frac{R}{p(\delta (A)|X)}I\left[D_{g}(X)\neq \delta (A)\right]\right]-E_{n}\left[\frac{R}{p(\delta (A)|X)}\right]$, where $E_{n}$ is the empirical average based on the training data. To alleviate the difficulty of the discontinuity and nonconvexity of the 0–1 loss in the treatment group-based weighted misclassification error, for each x∈𝒳 and a∈𝒜, we propose replacing the 0–1 loss function $I\left[D_{g}(x)\neq \delta (a)\right]$ by a angle-based loss $L_{\phi }(\delta (a),f(x))$, as proposed in Zhang and Liu (2014) and Zhang et al. (2016). The group-based decision rule $D_{g}$ is determined by the decision function f mapping from 𝒳 to $R^{K_{n}-1}$. Specifically, We encode the k-th treatment group as a vector $W_{k}\in R^{K_{n}-1}$ with

$$W_{k}=\left\{\begin{array}{l} {\left(K_{n}-1\right)}^{-1/2}1_{K_{n}-1},\;k=1,\\ -\left(1+\sqrt{K_{n}}\right)/{\left(K_{n}-1\right)}^{3/2}1_{K_{n}-1}+{\left(\frac{K_{n}}{K_{n}-1}\right)}^{1/2}e_{k-1},\;k=2,3,\ldots ,K_{n,} \end{array}\right.$$

where $1_{K_{n}-1}$ is a vector of ones of length $K_{n}-1$, and $e_{k}\in R^{K_{n}-1}$ is a vector with the k-th element equal to one, and zero elsewhere. Specifically, when $K_{n}=2$, we have $W_{1}=1$ and $W_{2}=-1$, which corresponds to the standard coding procedure in the binary classification problem. In addition, based on this coding procedure, one can check that, this treatment group simplex is symmetric with all vertices share an equal distance from each other in $R^{K_{n}-1}$. We refer Zhang and Liu (2014) for more details about the angle-based classification method. The RAMSVM-based loss consists of a convex combination of two loss functions

(8) $$L_{\phi }\left(\delta \left(a\right),f\left(x\right)\right):=\left(1-\gamma \right)\left[\sum_{k\neq \delta \left(a\right)}{\left(1+\langle W_{k},f\left(x\right)\rangle \right)}^{+}\right]+\gamma \left[{\left(K_{n}-1-\langle W_{\delta \left(a\right)},f\left(x\right)\rangle \right)}^{+}\right],$$

where γ∈[0,1]. The final group-based decision rule is obtained from

$$D_{g}(x)=\underset{k\in \left[K_{n}\right]}{arg\;max}\left\langle W_{k},f(x)\right\rangle .$$

The corresponding optimization problem is

(9) $$\underset{\delta ,f_{n}\in {ℱ}_{n}}{min}\;\left\{E_{n}\left[\frac{R}{p(\delta (A)|X)}L_{\phi }(\delta (A),f(X))\right]-K_{n}E_{n}\left[\frac{R}{p(\delta (A)|X)}\right]+\lambda_{n}∥f{∥}_{{ℱ}_{n}}^{2}\right\},$$

where ${ℱ}_{n}$ is a pre-specified function class of $\left\{f:𝒳\to R^{K_{n}-1}\right\}$, $\lambda_{n}$ is a tuning parameter, and $∥\cdot {∥}_{{ℱ}_{n}}$ is the functional penalty associated with ${ℱ}_{n}$ to overcome overfitting.

### 2.3. Implementation of GROWL

We introduce efficient algorithms to solve the optimization problem (9). To this end, we follow the procedure proposed by Liu et al. (2018) to replace R with the residual R-s(X). The rational is that removing the main effect that is independent of treatment should not affect the treatment decision while using residuals can significantly reduce the variability of weights to improve algorithm performance. However, note that the residual R-s(X) can take negative values, which would break the convexity of the minimization problem. In this case, we can switch the treatment group to other different treatment groups under the uniform sampling procedure. Specifically, it can be checked that, for any fixed δ, the following two optimization problems are equivalent:

(10) $$\begin{array}{l} \;\underset{f}{min}\;E\left[\frac{R\;-\;s(X)}{p(\delta (A)|X)}L_{\phi }(\delta (A),f(X))\right]⟺\underset{f}{min}\;E\left[\frac{(R\;-\;s(X){)}^{+}}{p(\delta (A)|X)}L_{\phi }(\delta (A),f(X))\right]\\ \;+\left(K_{n}-1\right)E\left[\frac{(R\;-\;s(X){)}^{-}}{p(\delta (A)|X)}L_{\phi }(\tilde{\delta }(A),f(X))\right], \end{array}$$

where $u^{+}=max(u,0),u^{-}=max(-u,0)$, and the conditional distribution of the random variable $\tilde{\delta }(A)$ is determined by $Pr(\tilde{\delta }(A)=k|\delta (A),X)=\frac{1}{K_{n}-1}$ for k≠δ(A) and 0 for k=δ(A). In this way, the weight term can be easily computed by $\frac{(R-s(X){)}^{+}}{p(\delta (A)|X)}+\left(K_{n}-\right.1)\;\frac{(R-s(X){)}^{-}}{p(\delta (A)|X)}$. For simplicity of notations, in the following of this section, we use $R^{⋆}$ to denote $(R-s(X){)}^{+}+\left(K_{n}-1\right)(R-s(X){)}^{-}$. The derivations of why the two optimization problems in (10) are equivalent can be seen in Appendix C.

Next we specify the decision function $f:𝒳\to R^{K_{n}-1}$ in a product Reproducing Kernel Hilbert Space (RKHS) ${ℱ}_{n}={⊗}_{k=1}^{K_{n}-1}{ℋ}_{\kappa }^{k}$. We develop efficient algorithms to solve (9) after replacing R with $R^{*}$ and switching treatments for observations with negative residuals. Our implementation consists of two steps. Step 1: under any fixed partition candidate δ, we convert the RAMSVM-based weighted classification problem (9) to a dual quadratic programming problem with box constraints. Then we solve the dual problem using coordinate descent algorithm to obtain the estimated optimal decision function under δ, denoted as ${\hat{f}}^{\delta }$; Step 2: Treatment partition estimation step: after plugging $\left(\delta ,{\hat{f}}^{\delta }\right)$ back into (9) to get the value (smaller is preferred) for the candidate δ, we propose to use the genetics algorithm (Goldberg and Holland, 1988), which is a stochastic search and evolutionary algorithm to obtain the optimal $\overset{ˆ}{\delta }$. Alternatively, we can also use the coordinate descent type of greedy algorithm to adjust the partition.

For step 1, we propose the following algorithm to solve the weighted classification problem when specifying f in the product linear space or product RKHS. Specifically, let $\omega_{i}=\frac{R_{i}^{*}}{p\left(\delta \left(A_{i}\right)|X_{i}\right)}$ be the weight for subject i∈[n]. For x∈𝒳, denote $f(x)={\left(f_{1}(x),\ldots ,f_{K_{n}-1}(x)\right)}^{T}$ and $W_{j,k}$ represents the k-th element of $W_{j}$, where $j\in \left[K_{n}\right]$ and $k\in \left[K_{n}-1\right]$.

For linear decision functions, we assume $f_{k}(x)=x^{T}\beta_{k}$ with $k\in \left[K_{n}-1\right]$, where $\beta_{k}$’s are our parameters of interest. The penalty term $∥f{∥}_{{ℱ}_{n}}^{2}=\sum_{k=1}^{K_{n}-1}\;\beta_{k}^{T}\beta_{k}$. Note that we include the intercepts in 𝒳 to simplify notation. After introducing slack variables for (9) and taking partial derivative of the Lagrangian function with respect to each $\beta_{k}$ and slack variables, we can derive the following dual problem with respect to the Lagrangian multiplier $\alpha_{i,j}$ and obtain ${\left({\hat{\alpha }}_{i,j}\right)}_{i\in [n];j\in \left[K_{n}\right]}$ by solving

(11) $$\begin{array}{l} \;\underset{{\left(\alpha_{i,j}\right)}_{i\in [n];j\in \left[K_{n}\right]}}{min}\;\frac{1}{2n\lambda }\sum_{k=1}^{K_{n}-1}\;\;{\left[\sum_{i=1}^{n}\;\;\alpha_{i,\delta \left(A_{i}\right)}W_{\delta \left(A_{i}\right),k}X_{i}-\sum_{i=1}^{n}\;\;\sum_{j\neq \delta \left(A_{i}\right)}\alpha_{i,j}W_{j,k}X_{i}\right]}^{T}\\ \;\left[\sum_{i=1}^{n}\alpha_{i,\delta \left(A_{i}\right)}W_{\delta \left(A_{i}\right),k}X_{i}-\sum_{i=1}^{n}\;\;\sum_{j\neq \delta \left(A_{i}\right)}\alpha_{i,j}W_{j,k}X_{i}\right]\\ \;-\sum_{i=1}^{n}\;\;\alpha_{i,\delta \left(A_{i}\right)}\left(K_{n}-1\right)-\sum_{i=1}^{n}\;\;\sum_{j\neq \delta \left(A_{i}\right)}\alpha_{i,j},\\ \;\text{s.t.}\;0⩽\alpha_{i,j}⩽\omega_{i}\left(\gamma I\left[j=\delta \left(A_{i}\right)\right]+\left(1-\gamma \right)I\left[j\neq \delta \left(A_{i}\right)\right]\right)\left(i\in \left[n\right];j\in \left[K_{n}\right]\right). \end{array}$$

Moreover, we can calculate

$${\hat{\beta }}_{k}=\frac{1}{n\lambda }\left[\sum_{i=1}^{n}\;{\overset{ˆ}{\alpha }}_{i,\delta \left(A_{i}\right)}W_{\delta \left(A_{i}\right),k}X_{i}-\sum_{i=1}^{n}\;\;\sum_{j\neq \delta \left(A_{i}\right)}{\overset{ˆ}{\alpha }}_{i,j}W_{j,k}X_{i}\right].$$

Note that one can verify that the quadratic optimization function in (11) is strictly convex with respect to each $\alpha_{i,j}$. The constraints in (11) are box constraints. Therefore, (11) can be solved efficiently by the well-known coordinate descent algorithm. Compared with standard Quadratic Programming (QP) algorithms for solving the dual problem, the coordinate descent algorithm can enjoy a faster computational speed and obtain more accurate solutions (Zhang et al., 2016). The final estimated group-based ITR is obtained by ${\hat{D}}_{g}(x)=arg\;{max}_{j\in \left[K_{n}\right]}\;\langle W_{j},\overset{ˆ}{f}(x)\rangle$, where $\overset{ˆ}{f}(x)={\left({\overset{ˆ}{f}}_{1}(x),\ldots ,{\hat{f}}_{K_{n}-1}(x)\right)}^{T}$ and ${\hat{f}}_{k}(x)=x^{T}{\hat{\beta }}_{k}$ for $k\in \left[K_{n}-1\right]$.

To deal with more complicated functions, we generalize the linear approach to obtain a nonlinear decision function in RKHS. To begin with, denote κ to be the corresponding kernel function and $G={\left(\kappa \left(X_{i},X_{i^{\prime }}\right)\right)}_{i,i^{\prime }\in [n]}$ to be the gram matrix. We assume G is invertible. Denote $G_{i}$ to be the i-th column of G. By using the $L_{2}$ norm in ${⊗}_{k=1}^{K_{n}-1}{ℋ}_{\kappa }^{k}$ for the penalty term, i.e., $∥f{∥}_{{ℱ}_{n}}^{2}=\sum_{k=1}^{K_{n}-1}\;\theta_{k}^{T}G\theta_{k}$, we can represent the decision function as $f_{k}(x)=\theta_{k,0}+\sum_{i=1}^{n}\;\theta_{k,i}\kappa \left(X_{i},x\right)$ for $k\in \left[K_{n}-1\right]$. Here, $\theta_{k}={\left(\theta_{k,1},\ldots ,\theta_{k,n}\right)}^{T}$ is our kernel product coefficient vector for $k\in \left[K_{n}-1\right]$. Similar to the steps in linear case, ${\left({\hat{\alpha }}_{i,j}\right)}_{i\in [n];j\in \left[K_{n}\right]}$ can be obtained by solving the following dual problem

(12) $$\begin{array}{l} \;\underset{{\left(\alpha_{i,j}\right)}_{i\in [n];j\in \left[K_{n}\right]}}{min}\;\frac{1}{2n\lambda }\sum_{k=1}^{K_{n}-1}\;\;{\left[\sum_{i=1}^{n}\;\;\alpha_{i,\delta \left(A_{i}\right)}W_{\delta \left(A_{i}\right),k}G_{i}-\sum_{i=1}^{n}\;\;\sum_{j\neq \delta \left(A_{i}\right)}\alpha_{i,j}W_{j,k}G_{i}\right]}^{T}\\ \;G^{-1}\left[\sum_{i=1}^{n}\;\;\alpha_{i,\delta \left(A_{i}\right)}W_{\delta \left(A_{i}\right),k}G_{i}-\sum_{i=1}^{n}\;\;\sum_{j\neq \delta \left(A_{i}\right)}\alpha_{i,j}W_{j,k}G_{i}\right]\\ \;+\frac{1}{2n\lambda }\sum_{k=1}^{K_{n}-1}\;\;{\left[\sum_{i=1}^{n}\;\;\alpha_{i,\delta \left(A_{i}\right)W_{\delta \left(A_{i}\right),k}}-\sum_{i=1}^{n}\;\;\sum_{j\neq \delta \left(A_{i}\right)}\alpha_{i,j}W_{j,k}\right]}^{2}\\ \;-\sum_{i=1}^{n}\;\;\alpha_{i,\delta \left(A_{i}\right)}\left(K_{n}-1\right)-\sum_{i=1}^{n}\;\;\sum_{j\neq \delta \left(A_{i}\right)}\alpha_{i,j},\\ \;\text{s.t.}\;0⩽\alpha_{i,j}⩽\omega_{i}\left(\gamma I\left[j=\delta \left(A_{i}\right)\right]+(1-\gamma )I\left[j\neq \delta \left(A_{i}\right)\right]\right)\left(i\in [n];j\in \left[K_{n}\right]\right). \end{array}$$

Furthermore, we can obtain

$$\begin{array}{l} {\hat{\theta }}_{k}=\frac{1}{n\lambda }G^{-1}\left[\sum_{i=1}^{n}\;\;{\hat{\alpha }}_{i,\delta \left(A_{i}\right)}W_{\delta \left(A_{i}\right),k}G_{i}-\sum_{i=1}^{n}\;\;\sum_{j\neq \delta \left(A_{i}\right)}{\hat{\alpha }}_{i,j}W_{j,k}G_{i}\right]\\ {\overset{ˆ}{\theta }}_{k,0}=\frac{1}{n\lambda }\left[\sum_{i=1}^{n}\;\;{\overset{ˆ}{\alpha }}_{i,\delta \left(A_{i}\right)}W_{\delta \left(A_{i}\right),k}-\sum_{i=1}^{n}\;\;\sum_{j\neq \delta \left(A_{i}\right)}\;{\hat{\alpha }}_{i,j}W_{j,k}\right]. \end{array}$$

One can check that (12) can be solved in an analogous manner as (11). The final decision function is obtained from ${\hat{f}}_{k}(x)={\hat{\theta }}_{k,0}+\sum_{i=1}^{n}\;{\overset{ˆ}{\theta }}_{k,i}\kappa \left(X_{i},x\right)$ for $k\in \left[K_{n}-1\right]$. More details about how the original problem (9) is transformed to the dual problems (11) and (12) in step 1 are provided in Appendix C.

For step 2, after we plug $\left(\delta ,{\hat{f}}^{\delta }\right)$ back to (9), we get the value for the candidate partition δ. We formulate the partition space as the discrete problem of partitioning $M_{n}$ numbers into $K_{n}$ groups. To solve this non-convex integer programming problem, when $M_{n}$ and $K_{n}$ are relatively small, we can implement the genetics algorithm using the R package called GA introduced in Scrucca (2013). Furthermore, if $K_{n}\ll M_{n}$ and both $K_{n}$ and $M_{n}$ are large, then the total number of partitions can be very large. Consequently, the genetics algorithm can be time consuming. Hence, to deal with this case, we propose a coordinate descent type of greedy algorithm to search for the optimal partition iteratively. Specifically, at each iteration, we minimize (9) by successively adjusting the group assignment for one specific treatment while holding the assignment of other treatments fixed. We go through each treatment in a cyclic fashion until convergence. The initial partition can be obtained via clustering the fitted conditional expected outcome for each treatment. The conditional expected outcome can be roughly estimated by $L_{1}$ penalized regression (Qian and Murphy, 2011), random forest or latent supervised clustering using the pairwise fusion penalty (Chen et al., 2021).

### 2.4. Selection of Treatment Group Number

Our analysis so far treats the group number $K_{n}$ as given. However, $K_{n}$ is typically unknown in practice. We propose the following effective procedure to determine $K_{n}$. We first randomly split the observed data ${\left\{\left(X_{i},A_{i},R_{i}\right)\right\}}_{i=1}^{n}$ into two folds. For each group number $1⩽K⩽M_{n}$, denote the ${\hat{\delta }}_{K}$ and ${\hat{D}}_{g,K}$ as the estimated optimal partition and associated group-based decision rule learned from one fold of the training data based on the implementations discussed in Section 2.3. Then, we calculate the estimated value function ${\hat{𝒱}}_{1}({\hat{\delta }}_{K},{\hat{D}}_{g,K})$ for each K using

(13) $${\hat{𝒱}}_{1}({\hat{\delta }}_{K},{\hat{D}}_{g,K})=\frac{E_{n}[RI[{\hat{D}}_{g,K}(X)\;=\;{\hat{\delta }}_{K}(A))/p({\hat{\delta }}_{K}(A)|X)]}{E_{n}[I[{\hat{D}}_{g,K}(X)\;=\;{\hat{\delta }}_{K}(A)]/p({\hat{\delta }}_{K}(A)|X)]},$$

where $E_{n}$ denotes the empirical mean of the other fold of observed data. Note that when K=1, all the treatments are grouped together and the associated ${\hat{𝒱}}_{1}({\hat{\delta }}_{1},{\hat{D}}_{g,1})$ corresponds to the value when we randomly recommend treatments. Thus, we can obtain the estimated benefit function of the estimated group-structured ITR for each K with

$$\begin{array}{l} {\hat{B}}_{en}(K)\;={\hat{𝒱}}_{1}({\overset{ˆ}{\delta }}_{K},{\hat{D}}_{g,K})-{\hat{𝒱}}_{1}({\hat{\delta }}_{1},{\hat{D}}_{g,1})\\ \;={\hat{𝒱}}_{1}({\hat{\delta }}_{K},{\hat{D}}_{g,K})-E_{n}[R]. \end{array}$$

We replicate the above process T times. For each replication t=1,2,…,T, denote the benefit function as ${\hat{B}}_{en}^{\left(t\right)}(K)$. We propose the following procedure that can be interpreted as the trade-off between the benefit and the variability of the estimated group-structured ITR to determine the optimal $K_{n}$:

(14) $${\hat{K}}_{n}=\underset{1<K⩽M_{n}}{arg\;max}\left\{\underset{t}{mean}\left[{\left\{{\hat{B}}_{en}^{\left(t\right)}(K)\right\}}_{t=1}^{T}\right]\right\}.$$

One can also replace R with $R-\hat{s}(X)$ in (13) to remove variability coming from estimating the main effect.

The group number estimator (14) can be interpreted as follows. Denote $\delta_{K}^{*}$ as the optimal partition when the group number is K. For $1⩽K⩽M_{n}$, let $B_{en}(K):=E_{X}\left[{max}_{k\in [K]}\;E\left[R|A\in G_{k}^{\delta_{K}^{*}},X\right]-R\right]$ be the maximum benefit when the group number is specified as K and $B_{en}^{*}:=E_{X}\left[{max}_{a\in 𝒜}\;E[R|X,A=a]-R\right]=B_{en}\left(M_{n}\right)$ be the optimal benefit. The optimal benefit $B_{en}^{*}$ corresponds to the case that we do not consider any group structure in the treatment space. We first consider the case that the treatments have homogeneous group structure ${𝒢}_{0}$ discussed in Section 2.2 and the true value of group number equals to $K_{0}$. Then, similar to the proof of Lemma 2 shown in Appendix C, one can check that if setting $K>K_{0}$, then the optimal partition defined in (7) would result in over identified group structures. These over identified optimal group structures can be any refinement of ${𝒢}_{0}$. In particular, these refined optimal partitions $\delta_{K}^{*}$’s of $\delta_{K_{0}}^{*}$ all lead to the same optimal benefit $B_{en}(K)=B_{en}^{*}$ when $K_{0}⩽K⩽M_{n}$. In this case, the bias of the value function is 0. However, the stochastic error bound and the convergence rate of the estimated value function shown in Theorem 6 in Section 3.3 increases with a polynomial rate $𝒪\left(K_{n}^{2}\right)$ as $K_{n}$ becomes larger. This demonstrates that as $K_{n}$ increases, the variability of the group-structured ITR becomes larger. Based on Xia et al. (2009), this variability is involved in (14) by using T times of sample splitting. Therefore, the group number selection procedure (14) incorporates the penalization of variability to avoid the over identified group structures when $K⩾K_{0}$ for the homogeneous case.

When the homogeneous case does not hold, then our optimal benefit $B_{en}(K)$ may be strictly less than the optimal benefit $B_{en}^{*}$ for all $1⩽K<M_{n}$. One can check that $B_{en}(K)$ is a non-decreasing function as K increases by induction, and finally equals to $B_{en}^{*}$ when $K=M_{n}$. For the non-homogeneous case, together with the same analysis of the increasing variability as K increases, the selection of group number can be interpreted as a trade-off between the benefit and the variability of the group-structured ITR.

#### Remark 3

*In practice, when the estimated optimal* ${\hat{K}}_{n}$ *is relatively small, we conclude that the gain of smaller variability would dominate the potential loss of the benefit for the group-structured ITR. Hence, a homogeneous or nearly-homogeneous group structure of the treatment effects is expected. In contrast, when* ${\hat{K}}_{n}$ *is close to* $M_{n}$, *the gain of higher benefit would dominate the negative effect of the large variability. For this scenario, we expect that the treatments may behave very differently from each other on the same patient. When* ${\hat{K}}_{n}=M_{n}$, *GROWL would learn the optimal ITR under the individual treatment domain without grouping. Its performance becomes similar to the traditional methods that do not consider the treatment structure*.

## 3. Theoretical Properties

In this section, we establish Fisher consistency of the optimal partition and associated group-based ITR for GROWL. We further obtain an excess risk bound and derive the convergence rate for the value function with the diverging group number $K_{n}$.

### 3.1. Fisher Consistency

Denote $\left(\delta_{\phi }^{*},f_{\phi }^{*}\right)$ as the optimal partition and associated optimal decision function under the generalized $L_{\phi }$ risk function ${\tilde{ℛ}}_{\phi }(\delta ,f)$:

(15) $$(\delta_{\phi }^{*},f_{\phi }^{*})\in \underset{\delta ,f:𝒳\to R^{K_{n}-1}}{\arg min}\left\{{\tilde{ℛ}}_{\phi }\left(\delta ,f\right)≔{ℛ}_{\phi }\left(\delta ,f\right)-K_{n}E\left[\frac{R}{p\left(\delta \left(A\right)|X\right)}\right]\right\},$$

where the $L_{\phi }$ risk function ${ℛ}_{\phi }(\delta ,f)$ is defined as

(16) $${ℛ}_{\phi }(\delta ,f):=E\left[\frac{R}{p(\delta (A)|X)}L_{\phi }(\delta (A),f(X))\right].$$

Let $f_{\phi }^{\delta }$ be the optimal decision function for any fixed $\delta :f_{\phi }^{\delta }=arg\;{min}_{f:𝒳\to R^{K_{n}-1}}\;{\tilde{ℛ}}_{\phi }(\delta ,f)$. Under the angle-based weighted classification framework, a classifier is said to be Fisher consistent if for each partition δ and x∈𝒳, the predicted treatment group has the maximum conditional group treatment effect under δ:

$$\underset{k\in \left[K_{n}\right]}{arg\;max}\langle W_{k},f_{\phi }^{\delta }(x)\rangle =\underset{k\in \left[K_{n}\right]}{arg\;max}\;E[R|A\in G_{k}^{\delta },X=x].$$

For our problem, we establish the generalized Fisher consistency results for both partition δ and the decision rule under the group domain if we choose $L_{\phi }$ to be the surrogate loss function, i.e., the derived optimal decision rule is the same as the one using the 0–1 loss. In particular, the following generalized Fisher consistency holds:

#### Theorem 4

*Let* $\delta^{*}$ *and* $\Delta^{*}$ *be defined in* (7) *and* $\left(\delta_{\phi }^{*},f_{\phi }^{*}\right)$ *be defined in* (15). *Denote* $D_{g}^{*}(x)$ *to be the optimal deterministic group-based ITR under* $\delta^{*}\in \Delta^{*}$, *which leads to the Bayesian rule* ${\text{arg}\;max}_{k\in \left[K_{n}\right]}\;E(R|A\in G_{k}^{\delta^{*}},X=x)$. *If* $\gamma \in \left[0,\frac{1}{2}\right]$, *then we have* $\delta_{\phi }^{*}\in \Delta^{*}$ *and* $arg\;{max}_{k\in \left[K_{n}\right]}\;\langle W_{k},f_{\phi }^{*}(x)\rangle =D_{g}^{*}(x)$.

### 3.2. Excess Risk

Next we establish the excess risk of 0–1 loss can be upper bounded by that of RAMSVM loss. To start with, we introduce the following notations. For any group-based ITR $D_{g}$, there exists a decision function $f:𝒳\to R^{K_{n}-1}$ such that $D_{g}(x)=arg\;{max}_{k\in \left[K_{n}\right]}\;\left\langle W_{k},f(x)\right\rangle$. Similar to the definition of $ℛ\left(\delta ,D_{g}\right)$, we define

$$ℛ\left(\delta ,f\right)=E\left[I\left[\underset{k\in \left[K_{n}\right]}{\text{arg}\;\text{max}}\langle W_{k},f\left(X\right)\rangle \neq \delta \left(A\right)\right]\frac{R}{p\left(\delta \left(A\right)|X\right)}\right],$$

and denote $\tilde{ℛ}(\delta ,f)=ℛ(\delta ,f)-E[R/p(\delta (A)|X)]$. Then the generalized Bayesian risk is denoted as ${\tilde{ℛ}}^{*}={inf}_{\delta ,f}\;\left\{\tilde{ℛ}(\delta ,f)|\delta ,f:𝒳\to R^{K_{n}-1}\right\}$. In terms of the value function ${𝒱}_{1}\left(\delta ,D_{g}\right)$ defined in (4), we observe that ${𝒱}_{1}(\delta^{*},D_{g}^{*})-{𝒱}_{1}(\delta ,D_{g})=\tilde{ℛ}(\delta ,f)-{\tilde{ℛ}}^{*}$. Note that in GROWL, we replace the 0–1 loss with the $L_{\phi }$ loss. Recall we have defined the generalized $L_{\phi }$ risk function ${\tilde{ℛ}}_{\phi }(\delta ,f)$ in (15) and (16). Similarly, the infimum of generalized $L_{\phi }$ risk function is defined as ${\tilde{ℛ}}_{\phi }^{*}={inf}_{\delta ,f}\;\left\{{\tilde{ℛ}}_{\phi }(\delta ,f)|\delta ,f:𝒳\to R^{K_{n}-1}\right\}$. In addition, under any fixed δ, let $f^{\delta }=arg\;{min}_{f:𝒳\to R^{K_{n}-1}}\;ℛ(\delta ,f)$ be the optimal decision function under the group domain. Denote $f^{*}=arg\;{min}_{f:𝒳\to R^{K_{n}-1}}\;ℛ(\delta^{*},f)$.

The following theorem shows the relationship between the generalized excess 0–1 risk $\tilde{ℛ}(\delta ,f)-{\tilde{ℛ}}^{*}$ and generalized excess $L_{\phi }$ risk ${\tilde{ℛ}}_{\phi }(\delta ,f)-{\tilde{ℛ}}_{\phi }^{*}$ under some bounded restrictions for f.

#### Theorem 5 (Bound for excess risk)

*For any partition* δ, *any measurable function* $f\;:𝒳\to R^{K_{n}-1}$ *such that* $\left\langle W_{k},f(x)\right\rangle \in \left[-1,K_{n}-1\right]$ *holds for* ∀x∈𝒳 *and* $\forall k\in \left[K_{n}\right]$, *and any probability distribution for* (X,A,R), *we have*

$$\tilde{ℛ}(\delta ,f)-{\tilde{ℛ}}^{*}⩽{\tilde{ℛ}}_{\phi }(\delta ,f)-{\tilde{ℛ}}_{\phi }^{*}.$$

Note that Theorem 5 is different from Theorem 3.2 in Zhao et al. (2012) in the sense that we consider multiple treatments, and dealing with both partition δ and the decision function.

### 3.3. Convergence Rate

Define the estimated optimal partition ${\hat{\delta }}_{n}$ and group-based decision function ${\hat{f}}_{n}$ as

$$({\overset{ˆ}{\delta }}_{n},{\overset{ˆ}{f}}_{n})\in \underset{\delta ,f\in {⊗}_{k=1}^{K_{n}-1}{ℋ}_{\kappa }^{k}}{arg\;min}\left\{E_{n}\left[\frac{R}{p(\delta (A)|X)}L_{\phi }(\delta (A),f(X))-K_{n}\frac{R}{p(\delta (A)|X)}\right]+\lambda_{n}∥f{∥}_{{ℱ}_{n}}^{2}\right\}.$$

For a fixed partition δ, denote the optimal estimated group-based decision function as

$${\hat{f}}_{n}^{\delta }=\underset{f\in {⊗}_{k=1}^{K_{n}-1}{ℋ}_{\kappa }^{k}}{arg\;min}\left\{E_{n}\left[\frac{R}{p(\delta (A)|X)}L_{\phi }(\delta (A),f(X))-K_{n}\frac{R}{p(\delta (A)|X)}\right]+\lambda_{n}∥f{∥}_{{ℱ}_{n}}^{2}\right\}.$$

Specifically, for the decision function class, we restrict our consideration to the product RKHS associated with Radial Basis Function (RBF) kernels:

$$\kappa \left(x,x^{\prime }\right)=exp(-\sigma_{n}^{2}∥x-x{∥}^{2}),x,x^{\prime }\in 𝒳,$$

where $\sigma_{n}>0$ is a bandwidth parameter varying with n. For theoretical convenience, we assume ${\hat{f}}_{n}$ satisfies the extra bounded constraint $\langle W_{k},{\hat{f}}_{n}(x)\rangle \in \left[-1,K_{n}-1\right]$ for ∀x∈𝒳 and $\forall k\in \left[K_{n}\right]$. This constraint does not show up in the algorithm discussed in Section 2.3 because it makes the computation algorithm in Section 2.3 become more complicate and inefficient. Our numerical experience suggests that removing the constraint for ${\hat{f}}_{n}$ can yield better classification performance than including it.

Next we show that $\tilde{ℛ}({\hat{\delta }}_{n},{\hat{f}}_{n})$ converges to ${\tilde{ℛ}}^{*}$ and equivalently, the value function ${𝒱}_{1}({\hat{\delta }}_{n},{\hat{D}}_{g,n})$ converges to ${𝒱}_{1}(\delta^{*},D_{g}^{*})$ where the estimated group-based ITR ${\hat{D}}_{g,n}(x):=arg\;{max}_{k\in \left[K_{n}\right]}\;\langle W_{k},{\hat{f}}_{n}(x)\rangle$. We start with introducing the following quantity:

$${𝒜}_{\sigma_{n}}^{\delta }\left(\lambda_{n}\right)=\underset{f\in {⊗}_{k=1}^{K_{n}-1}{ℋ}_{\kappa }^{k}}{inf}\;\left\{\lambda_{n}∥f{∥}_{\kappa }^{2}+{\tilde{ℛ}}_{\phi }(\delta ,f)-{\tilde{ℛ}}_{\phi }(\delta ,f^{\delta })\right\}.$$

For a fixed δ, the term ${𝒜}_{\sigma_{n}}^{\delta }\left(\lambda_{n}\right)$ describes how well the regularized RAMSVM-risk approximates the optimal RAMSVM-risk in the RKHS. This quantity is often referred as the approximation error term (Steinwart and Scovel, 2007). Specifically, when $K_{n}=2$, Steinwart and Scovel (2007) proposed a geometric noise assumption to upper bound ${𝒜}_{\sigma_{n}}^{\delta }\left(\lambda_{n}\right)$ in the context of hinge loss based SVM classification problem under any fixed δ. In this paper, we generalize the geometric noise assumption so that we can upper bound ${𝒜}_{\sigma_{n}}^{\delta }\left(\lambda_{n}\right)$ for the multicategory group-based ITR problem under the RAMSVM-based loss. Under each δ, denote the difference of two group treatment effects as

$$\eta_{i,j}^{\delta }(x)=E[R|A\in G_{i}^{\delta },X=x]-E[R|A\in G_{j}^{\delta },X=x],\;i\neq j\;\text{and}\;i,j\in \left[K_{n}\right].$$

Define the decision regions for each pair of treatment groups $i,j\in \left[K_{n}\right]$ to be ${𝒪}_{ij}^{\delta ,-}=\{x\in \;𝒳|\eta_{i,j}^{\delta }(x)<0\}$ and ${𝒪}_{ij}^{\delta ,+}=\{x\in 𝒳|\eta_{ij}^{\delta }(x)>0\}$. Then let ${𝒳}_{i}^{\delta }={⋂}_{j\neq i}\;{𝒪}_{ij}^{\delta ,+}$ for $i\in [K_{n}]$ be the subset of 𝒳 where the treatment effect of group i dominates any other treatment groups under partition δ. Denote the function $\eta^{\delta }(x)=\sum_{i=1}^{K_{n}}\;I\left[x\in {𝒳}_{i}^{\delta }\right]{sup}_{j\neq i}\;|\eta_{i,j}^{\delta }(x)|$ as the maximum difference of the group treatment effects for each region ${𝒳}_{i}^{\delta }$. Furthermore, denote the following distance function to the decision boundary as $\Delta^{\delta }(x)=\sum_{i=1}^{K_{n}}\;I[x\in {𝒳}_{i}^{\delta }]{inf}_{j\neq i}\;dist(x,{𝒳}_{j}^{\delta })$, where dist(x,𝒪) is the distance between a point x and a set 𝒪. Then we define the following generalized geometric noise assumption:

#### Assumption 1 (Generalized geometric noise assumption)

*For a fixed* δ *and* $i\in \left[K_{n}\right]$, *there exists a constant* U>0 *such that for any* t>0, *we have*

$$E_{X}\left[exp(-\frac{{\left(\Delta^{\delta }(X)\right)}^{2}}{t})\left|\eta^{\delta }(X)\right|\right]⩽Ut^{qd/2},$$

*where* d *is the dimension of* 𝒳 *and* q *serves as the geometric noise exponent*.

One can check that when $K_{n}=2$, Assumption 1 is consistent with Definition 2.3 in Steinwart and Scovel (2007) and Definition 3.8 in Zhou et al. (2017). In some sense, this geometric noise exponent q describes the concentration of the measure $\left|\eta^{\delta }(x)\right|dP_{X}$ near the decision boundary. In the case of complete separation, i.e., $\eta^{\delta }(x)>\varphi_{0}>0$ for some constant $\varphi_{0},q$ can be as large as possible.

Let $\Gamma_{n}$ be the total number of partitions. Recall the definition of ${𝒱}_{1}^{*}(\delta )$ and $\Delta^{*}$ in (6) and (7). Consider $K_{n}$ can diverge to infinity as the sample size n increases. Then, for any n, there exist a positive gap $\Psi_{n}:=\frac{1}{2}{inf}_{\delta \notin \Delta^{*}}\;\{{𝒱}_{1}^{*}(\delta^{*})-{𝒱}_{1}^{*}(\delta )\}>0$, such that $\tilde{ℛ}(\overset{‾}{\delta },f^{\overset{‾}{\delta }})-{\tilde{ℛ}}^{*}={𝒱}_{1}^{*}(\delta^{*})-{𝒱}_{1}^{*}(\overset{‾}{\delta })>\Psi_{n}$ holds for any non-optimal partition $\overset{‾}{\delta }\notin \Delta^{*}$. Here, $\Psi_{n}$ can be interpreted as the signal to characterize the minimum distance of the value function between the optimal partitions and any other non-optimal partitions. Intuitively, we need the signal $\Psi_{n}$ to be large enough so that we can distinguish the optimal partitions from non-optimal partitions. Now we are ready to present the main theorem for the convergence rate of GROWL.

#### Theorem 6 (Convergence rate of ${\hat{\delta }}_{n}$ and value function for diverging $M_{n},K_{n}$)

*Suppose the generalized geometric noise assumption holds for an optimal partition* $\delta^{*}\in \Delta^{*}$ *with exponent* 0<q<∞ *and constant* U. *Further assume* $|\frac{R}{p\left(\delta^{*}(A)|X\right)}|⩽Z_{n}$ *and* $\theta (𝒳)⩽M_{1}$, *where* $\left\{Z_{n}\right\}$ *is a sequence, and* θ(𝒳) *is denoted as the volume of* 𝒳. *Let* $\epsilon_{n}=K_{n}^{1/2}Z_{n}^{1/2}n^{-1/2}\lambda_{n}^{-1/2}+K_{n}Z_{n}n^{-1/2}+K_{n}Z_{n}({\left(\lambda_{n}\right)}^{-\frac{2}{2+v}+\frac{\left(2-v)(1+\theta \right)}{\left(2+v)(1+q\right)}}n^{-\frac{2}{2+v}}+n^{-1}\lambda_{n}^{-1}+\lambda_{n}^{\frac{q}{q+1}})$. *Then, if* $M_{n}K_{n}^{2}/log\;n\to 0,n\lambda_{n}\to \infty ,\Psi_{n}/\epsilon_{n}\to \infty$ *hold for any* θ>0,0<v<2, *and take* $\sigma_{n}=\lambda_{n}^{-1/(q+1)d}$, *the following results hold:*

*(I) For any* θ>0,0<v<2, *we have*

$$Pr({\hat{\delta }}_{n}\in \Delta^{*})=1-𝒪\left(\Gamma_{n}\epsilon_{n}\right);$$

*(II)* $\tilde{ℛ}({\hat{\delta }}_{n},{\hat{f}}_{n})-{\tilde{ℛ}}^{*}={𝒪}_{p}\left(\Gamma_{n}\epsilon_{n}\right).$

For Theorem 6, we can choose $\lambda_{n}=n^{-\frac{q+1}{3q+1}}$, and let (θ,v) be sufficiently small. When data are well separated under one of the optimal partitions, q can be sufficiently large. Note that if the group-based propensity score $p(\delta^{*}(A)|X)$ has balanced structure under $\delta^{*}$, then as $K_{n}\to \infty ,p(\delta^{*}(A)|X)$ would decay uniformly. Thus, we have $|\frac{R}{p(\delta^{*}(A)|X)}|⩽Z_{n}=𝒪\left(K_{n}\right)$. In this case, the convergence rate for the value function can achieve ${𝒱}_{1}(\delta^{*},D_{g}^{*})-{𝒱}_{1}({\hat{\delta }}_{n},{\hat{D}}_{g,n})=\tilde{ℛ}({\hat{\delta }}_{n},{\hat{f}}_{n})-{\tilde{ℛ}}^{*}={𝒪}_{p}(\Gamma_{n}K_{n}^{2}n^{-\frac{1}{3}})$.

Note that $\epsilon_{n}$ in Theorem 6 describes the rate of the stochastic error bound for the regret. Thus, the assumption $\Psi_{n}/\epsilon_{n}\to \infty$ implies that, the signal $\Psi_{n}$ should be large enough and dominates the noise $\epsilon_{n}$ so that ${\hat{\delta }}_{n}$ can finally belong to $\Delta^{*}$. To better under illustrate this assumption, we consider the following simple example. Suppose the number of treatment groups is fixed. Then, under the homogeneous case, the best partition among the partition set without the optimal partitions corresponds to the case that only one treatment is misclustered. In this case, one can check that the defined signal term $\Psi_{n}=𝒪\left(1/M_{n}\right)$ based on the proof of Lemma 2. Hence, due to $M_{n}=o(log\;n)$, the assumption $\Psi_{n}/\epsilon_{n}\to \infty$ is satisfied since $\epsilon_{n}$ decays at a polynomial rate of n.

The pipeline for proving Theorem 6 is stated as the follow two steps: First, for any optimal partition $\delta^{*}\in \Delta^{*}$, we establish a finite sample bound for the difference between the expected outcome using the estimated group-based decision function ${\hat{f}}_{n}^{\delta^{*}}$ based on the training data and that of the optimal group-based decision function $f^{\delta^{*}}$ under $\delta^{*}$; Second, due to $\Psi_{n}/\epsilon_{n}\to \infty$, as n goes to infinity, the stochastic error $\epsilon_{n}$ of $\tilde{ℛ}(\overset{‾}{\delta },f^{\overset{‾}{\delta }})$ arising from using $E_{n}$ to estimate E in (17) would be dominated by the gap $\Psi_{n}$ for any $\overset{‾}{\delta }\notin \Delta^{*}$. Hence, ${\hat{\delta }}_{n}$ in (17) would finally belong to $\Delta^{*}$ when n is sufficient large since $\delta^{*}\in \Delta^{*}$ maximizes the value function ${𝒱}_{1}$. Then, the convergence rate is determined by the rate of $Pr({\hat{\delta }}_{n}\in \Delta^{*})\to 1$ and the convergence rate of the first step when treating the partition is fixed as $\delta^{*}\in \Delta^{*}$. Specifically, the novelty of our technical proof arises from bounding the approximation bias term ${𝒜}_{\sigma_{n}}^{\delta }\left(\lambda_{n}\right)$ with order $𝒪(K_{n}^{2}\lambda_{n}^{q/(q+1)})$ and deriving the finite value reduction bound in a multicategory setting. The intermediate results deriving from the first step generalize Lemma 3.9 in Zhou et al. (2017) and Theorem 3.4 in Zhao et al. (2012) from binary treatments to multiple treatment groups $K_{n}$ that may diverge to infinity as the sample size increases. More details are provided in Appendix B.

For the case that the number of treatments $M_{n}$ and treatment groups $K_{n}$ are fixed, it is straightforward to derive the following Corollary 7 from Theorem 6. Note that for the fixed group number case, $\Psi_{n}/\epsilon_{n}\to \infty$ is trivially satisfied since $\Psi_{n}$ is a constant.

#### Corollary 7 (Convergence rate of ${\hat{\delta }}_{n}$ and value function for fixed $M_{n}$ and $K_{n}$)

*Suppose the generalized geometric noise assumption holds for an optimal partition* $\delta^{*}\in \Delta^{*}$ *with exponent* 0<q<∞ *and constant* U. *Further assume* $|\frac{R}{p(\delta^{*}(A)|X)}|⩽Z_{0}$ *and* $\theta (𝒳)⩽M_{1}$ *where* $Z_{0}$ *and* $M_{1}$ *are constants. Then, if* $n\lambda_{n}\to \infty$, *and take* $\sigma_{n}=\lambda_{n}^{-1/(q+1)d}$, *the following results hold:*

*(I) For any* θ>0,0<v<2, *we have*

$$\mathrm{Pr}\left({\hat{\delta }}_{n}\in \Delta^{*}\right)=1-𝒪\left({\left(n\lambda_{n}\right)}^{-\frac{1}{2}}+{\left(\lambda_{n}\right)}^{-\frac{2}{2+v}+\frac{\left(2-v\right)\left(1+\theta \right)}{\left(2+v\right)\left(1+q\right)}}n^{-\frac{2}{2+v}}+\lambda_{n}^{\frac{q}{q+1}}\right);$$

*(II)* $\tilde{ℛ}({\hat{\delta }}_{n},{\hat{f}}_{n})-{\tilde{ℛ}}^{*}={𝒪}_{p}\left({\left(n\lambda_{n}\right)}^{-1/2}+{\left(\lambda_{n}\right)}^{-\frac{2}{2+v}+\frac{\left(2-v)(1+\theta \right)}{\left(2+v)(1+q\right)}}n^{-\frac{2}{2+v}}+\lambda_{n}^{\frac{q}{q+1}}\right).$

## 4. Simulation Studies

We evaluate the finite-sample performance of our proposed method using several simulation studies.

### 4.1. Homogeneous Settings

In this simulation study, we consider the setting where the treatment responses for the treatments in the same group are equivalent, but differ for the treatments across different groups. We generate 10-dimensional independent prognostic variables $X_{1},\ldots ,X_{10}$, following U[-1,1]. The outcome R is normally distributed with $E[R|A,X]=1+2X_{1}+X_{2}+0.5X_{3}+T_{0}(X,A)$ and standard deviation 1, where $T_{0}(X,A)$ reflects the interaction between the treatment and the prognostic variables. In addition, we assume that the treatment effects have the homogeneous grouping structure ${𝒢}^{0}$ induced by $\delta^{0}$ discussed in Section 2.2. Specifically, we consider the following three scenarios:

#### Scenario 1.

$M_{n}=10,K_{n}=2,{𝒢}^{0}=\{\{1,\;2,3,\;4,5\},\{6,\;7,8,\;9,10\}\}$ and $T_{0}(X,A)=1.8(0.2-\left.X_{1}-X_{2}\right)(I[A\in \{1,\;2,3,\;4,5\}]\times (-1)+I[A\in \{6,7,8,\;9,10\}]\times 1);$

#### Scenario 2.

$M_{n}=10,K_{n}=2,{𝒢}^{0}=\{\{1,\;2,3,\;4,5\},\{6,\;7,8,\;9,10\}\}$ and $T_{0}(X,A)=3.5(0.8-\left.X_{1}^{2}-X_{2}^{2}\right)(I[A\in \{1,\;2,3,\;4,5\}]\times (-1)+I[A\in \{6,7,8,\;9,10\}]\times 1);$

#### Scenario 3.

$M_{n}=15,K_{n}=3,{𝒢}^{0}=\{\{1,\;2,3,\;4,5\},\{6,\;7,8,\;9,10\},\{11,\;12,\;13,\;14,\;15\}\}$ and $T_{0}\left(X,A\right)=5\left(\left(-0.2+X_{1}+2X_{2}\right)I\left[A\in \left\{1,\;2,3,\;4,5\right\}\right]+\left(0.3+2X_{1}+X_{2}\right)I\left[A\in \left\{6,\;7,8,\;9,10\right\}\right]+\right.\left.\left(-0.2+3X_{1}\right)I[A\in \{11,\;12,\;13,\;14,\;15\}]\right)$.

Scenario 1 corresponds to 10 treatment arms belonging to two treatment groups with underlying linear decision boundaries whereas Scenario 2 considers the circle decision boundary. Scenario 3 includes 15 treatments compared with the first two and deals with three treatment groups with linear decision boundary. Since our studies are especially interested in the case that the propensity score of some specific treatments may be very small, we perform the following two designs varying from balanced to unbalanced designs within each scenario:

- Balanced Design: $p(a|x)=\frac{1}{M_{n}}$ for each $a\in 𝒜=\left[M_{n}\right]$ and each x∈𝒳;
- Unbalanced Design: The value of p(a∣x) for some specific treatments can be very small compared with other treatments.

Under the unbalanced design, for each x∈𝒳, the propensity scores for the first two scenarios are set to be $\left(\left(\frac{1}{20},\frac{1}{20},\frac{1}{20},\frac{3}{20},\frac{1}{5}\right),\left(\frac{1}{20},\frac{1}{20},\frac{1}{10},\frac{1}{10},\frac{1}{5}\right)\right)$ while the propensity scores equal to $\left(\left(\frac{1}{20},\frac{1}{20},\frac{1}{20},\frac{11}{120},\frac{11}{120}\right),\left(\frac{1}{20},\frac{1}{20},\frac{1}{20},\frac{11}{120},\frac{11}{120}\right),\left(\frac{1}{20},\frac{1}{20},\frac{7}{90},\frac{7}{90},\frac{7}{90}\right)\right)$ for Scenario 3. In addition, we conduct more simulation settings when the propensity scores are more unbalanced and may depend on the covariates. These additional results are shown in Appendix D. For GROWL, we use the linear kernel for Scenarios 1 and 3 and utilize the Gaussian kernel for Scenario 2 corresponding to different shapes of the decision boundary. The tuning parameter $\lambda_{n}$ is chosen to maximize the empirical value function $E_{n}[RI[\hat{D}(X)=A]/p(A|X)]/E_{n}[I[\overset{ˆ}{D}(X)=A]/p(A|X)]$ by 10-fold cross-validation among $\left\{\frac{1}{16},\frac{1}{8},\frac{1}{4},\frac{1}{2},1,\;2,4,\;8,16\right\}$. For the Gaussian kernel, we fix the inverse bandwidth of the kernel $\sigma_{n}$ with $1/\left(2{\hat{\tau }}^{2}\right)$, where $\hat{\tau }$ is the median of the pairwise Euclidean distance of the covariates (Wu and Liu, 2007). The treatment group number is determined by the trade-off procedure (14) with T=50 shown in Section 2.4.

The following four methods are compared under each scenario:

- SL: Super Learner based Q-learning method to estimate E[R∣X,A] (Polley and Van Der Laan, 2010);
- AD: Multi-armed Angle-based Direct learning using linear terms for Scenarios 1 and 3 and polynomial terms for Scenario 2 (Qi et al., 2020);
- PLS: $L_{1}$-Penalized Least Squares method (Qian and Murphy, 2011), which estimates E[R∣X,A] using the basis sets (1,X,A,XA) for Scenarios 1 and 3, and $\left(1,X^{2},A,X^{2}A\right)$ for Scenario 2;
- GROWL: Our proposed method.

SL aims to find the optimal combination of multiple estimated Q-functions by minimizing the cross-validated risk. For the collection of learning algorithms, we include ridge regression (Hoerl and Kennard, 1970), elastic net (Zou and Hastie, 2005), random forest (Breiman, 2001), XGBoosting (Chen and Guestrin, 2016), and neural network (Venables and Ripley, 2013). We refer Polley and Van Der Laan (2010) for more implementation details about SL. In addition, the tuning parameters in AD and PLS are selected to maximize $E_{n}[RI[\overset{ˆ}{D}(X)=A]/p(A|X)]/E_{n}[I[\overset{ˆ}{D}(X)=A]/p(A|X)]$ by 10-fold cross-validation.

We evaluate the above methods using the empirical value function and the group-based misclassification rate between the estimated group decision rules and the true group decision rules on an independently generated testing data of size 10,000. The empirical value function is calculated by the mean of treatment effects under the empirical distribution of X based on the estimated decision rule. Note that under the homogeneous setting, the maximum group-based treatment effect equals to the maximum individual treatment based effect. Hence, the misclassification rate under the group domain is equivalent to the misclassification rate under the individual treatment domain. For each scenario, the training sample sizes vary from 200, 400 to 600 and we replicate the simulations for 200 times.

**Figure 1:**
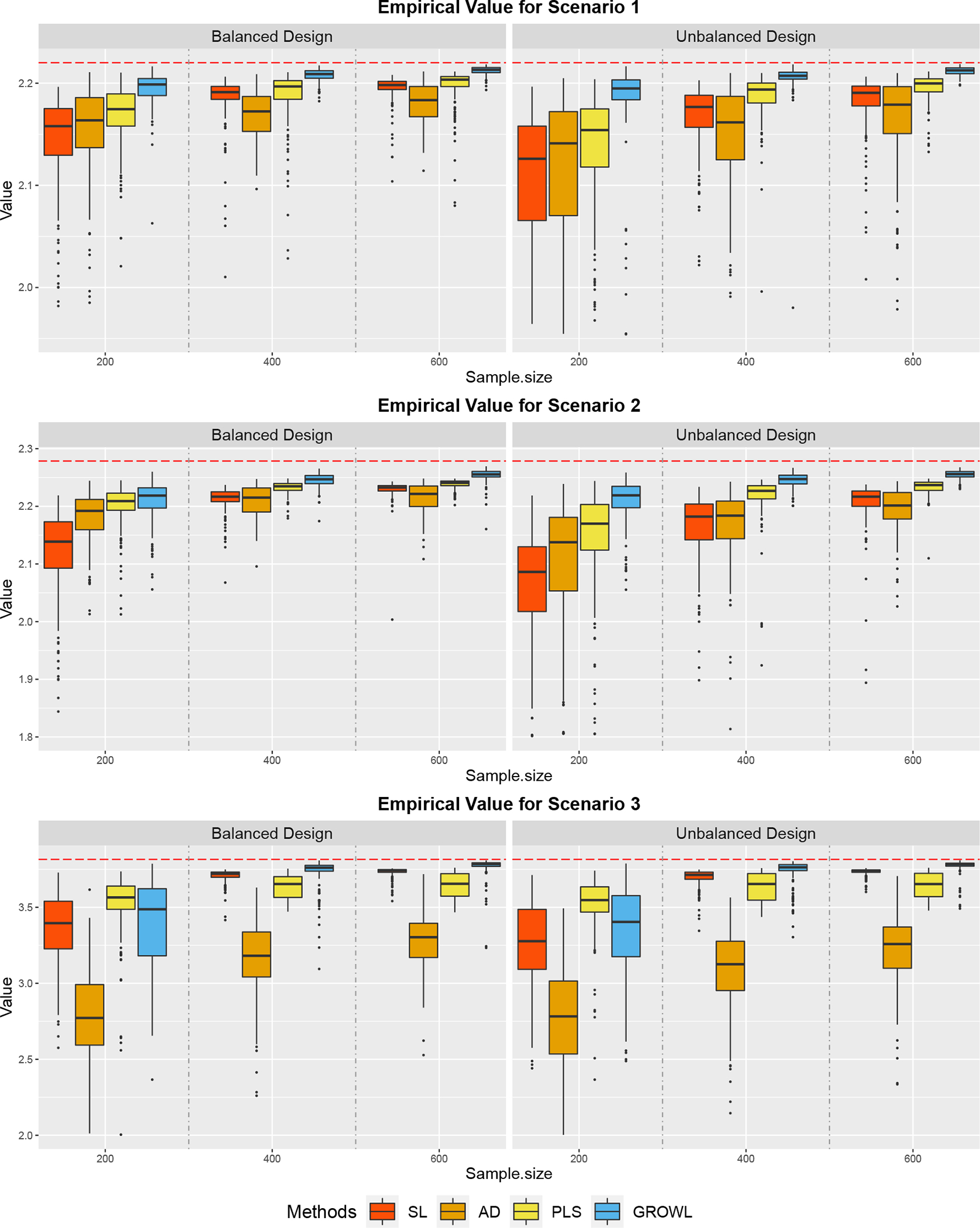
Boxplots of Empirical Value Function evaluated on the independent test data under the **homogeneous** settings. Red horizontal dashed lines show oracle values for each scenario.

We present the empirical value function of each scenario under different designs using boxplots in Figure 1. Results of group-based misclassification rates are included in Figure 5 of Appendix D. We also report the square root of Mean Square Error (MSE) of the empirical value function and the misclassification rate in Table 1. Based on Lemma 2 and Theorem 4, we have shown that $\delta^{0}$ should equal to the optimal partition $\delta^{*}$ defined in (7), and $\delta^{*}$ should equal to the optimal partition $\delta_{\phi }^{*}$ derived from (15). Accordingly, the Ratio column in Table 1 reports the ratio of our estimated ${\hat{\delta }}_{n}$ exactly being $\delta^{0}$ among the 200 replications. It can be seen that ${\hat{\delta }}_{n}$, the estimation of $\delta_{\phi }^{*}$, converges to $\delta^{0}$ with a high ratio as n becomes larger, which confirms part (I) of Theorem 6. In general, as the trial design becomes more unbalanced, all these methods perform worse, in the sense that MSE becomes larger for each scenario. Without considering the group structure for the treatments, SL, PLS and AD suffer from the inaccurate estimation of functions related to individual-treatment effects because of the small amount of observations for some specific treatments. However, GROWL estimates the group-structured ITR, which reduces the dimension of the treatment space and clusters the treatments that employ similar treatment effects into the same group. In addition, since GROWL estimates the ITR in the treatment group domain, the variance of the value function shrinks quicker than other methods as the training sample size increases. As is demonstrated in Figure 1 and Table 1, our method outperforms other methods in most cases with higher empirical value functions, smaller misclassfication rates, and especially lower variabilities for both evaluation criteria.

### 4.2. Non-homogeneous Settings

In many cases, it is possible that the treatment effects do not have exactly homogeneous grouping structure ${𝒢}^{0}$ assumed in Section 4.1. In this section, we perform nearly homogeneous and nonhomogeneous scenarios to examine our method. Specifically, we generalize Scenario 1 in Section 4.1 with the following Scenario 4 indexed by a parameter θ>0:

#### Scenario 4:

$M_{n}=10,\;T_{0}\left(X,A\right)=1.8\left(0.2-X_{1}-X_{2}\right)\left(I[A\in \{1,\;2,3,\;4,5\}]\times (-1-\frac{A}{\theta })+I[A\in \right.\left.\left\{6,\;7,8,\;9,10\}]\times (1+\frac{A-5}{\theta }\right)\right)$

The parameter θ determines the level of heterogeneity of treatment effects. As θ becomes smaller, the treatment effects become more diverse and thus the group structure tends to disappear. When θ=+∞, Scenario 4 has the exact homogeneous structure ${𝒢}^{0}=\{\{1,\;2,3,\;4,5\},\{6,\;7,8,9,10\}\}$ shown in Scenario 1. We vary θ from 40, 20, 10 to 5. In particular, the simulation scenarios in this section only focus on the unbalanced design. For GROWL, the treatment group number is determined by the trade-off procedure in (14). Other simulation settings and comparison methods are the same as those in Section 4.1.

**Figure 2:**
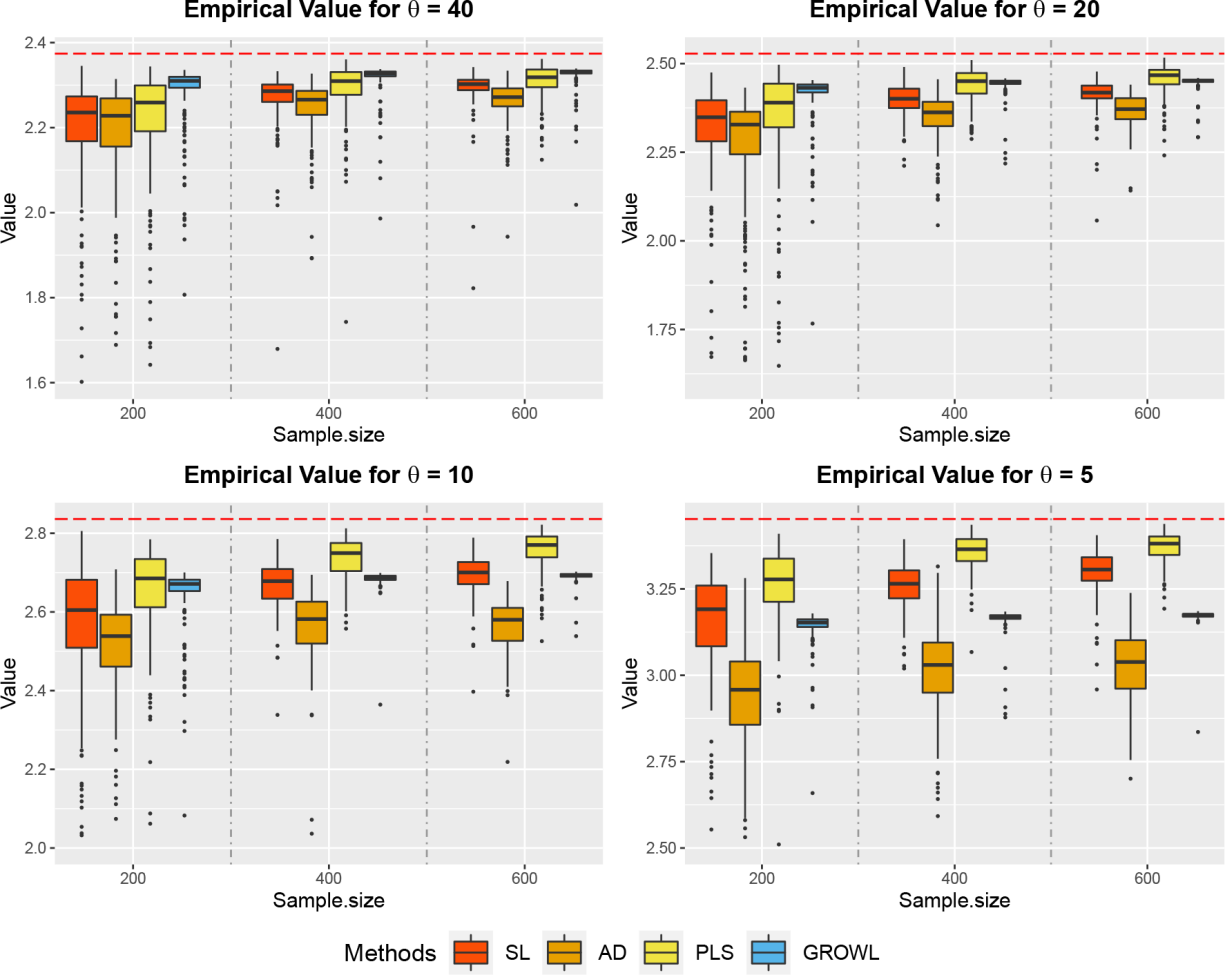
Boxplots of Empirical Value Function evaluated on the independent test data under the **nonhomogeneous** settings and unbalanced design. Red horizontal dashed lines show oracle values for each scenario.

Similar to Section 4.1, we provide boxplots of the empirical value function in Figure 2, and MSE of the empirical value function in Table 2. When θ decreases from 40 to 5, the treatment effects vary from nearly homogeneous structure to nonhomogeneous structure. For nearly homogeneous cases with θ=40 and 20, our method still outperforms other methods while for nonhomogeneous cases with θ=10 and 5, PLS performs the best. The results are consistent with Remark 3. For each $1⩽K_{n}<10$, the maximum benefit of group-structured ITR $B_{en}\left(K_{n}\right)$ is strictly less than the optimal benefit $B_{en}^{*}$ because the conditional treatment effects within the same group are different under $\delta_{K}^{*}$ in nonhomogeneous cases. When θ=+∞, these two values are equal. As θ decreases, the gap between these two values increases. Based on our simulation results, for each θ and training sample size, over 95% of the replications suggest ${\hat{K}}_{n}=2$ based on the trade-off procedure (14), and over 90% of the estimated partition still equals to the same two-group structure ${𝒢}^{0}=\{\{1,\;2,3,\;4,5\},\{6,\;7,8,\;9,10\}\}$ as the homogeneous setting. Hence, in Scenario 4, GROWL tends to sacrifice the benefit while retain small variability of the value function, and the gain of small variability continues to dominate the loss of benefit when θ decreases from 40 to 5. In Figure 2, one can observe that the empirical value of our method would not converge to the optimal value (shown by dashed lines) and a positive gap would exist. In addition, when sample size increases from 200 to 600, the relative improvement ratio in terms of the MSE for GROWL decreases when θ has smaller values. However, due to the usage of the group structure, the variability of our method is very small compared with others shown in Figure 2. Therefore, the trade-off between the benefit and variability of the value function for the group-structured ITR estimated by GROWL is clear. From Table 2, GROWL is still competitive in nonhomogeneous settings in terms of the MSE criterion.

## 5. Application to the STAR*D Study

In this section, we apply our proposed GROWL to analyze the data from the STAR*D study (Rush et al., 2004). The STAR*D study performed research on outpatients with nonpsychotic major depressive disorder. The goal of the study was to compare various treatment options for the patients who failed to obtain a satisfactory response with citalopram (CIT), an initial antidepressant treatment. The primary outcome was measured by the Quick Inventory of Depressive Symptomatology (QIDS) score ranging from 0 to 27, where higher scores indicate more severe depression.

**Figure 3:**
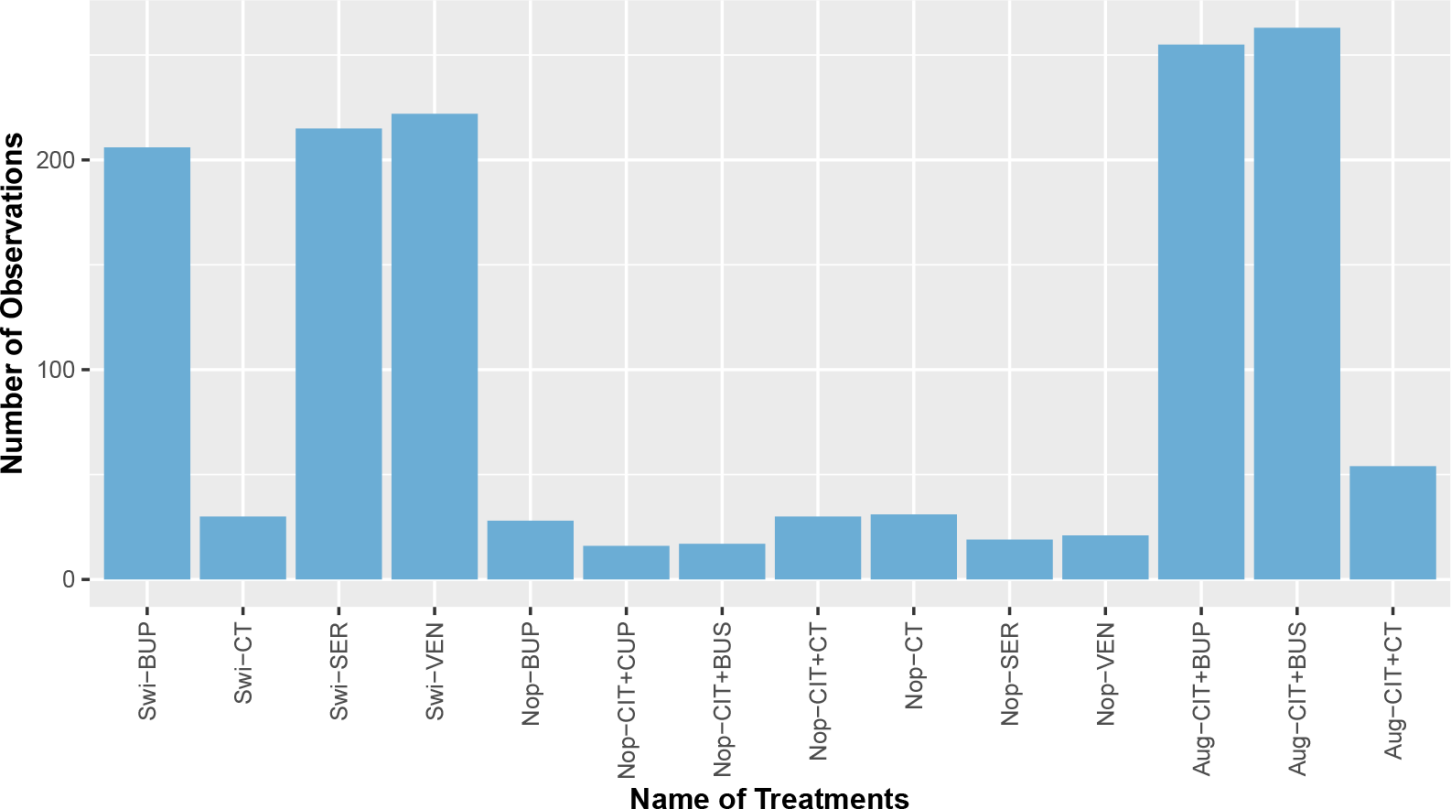
Distribution of observations for the 14 treatment options in the STAR*D dataset. “Swi”, “Aug” and “Nop” correspond to patients that switch to other treatments, augment existing treatments, and have no preference for two previous options.

The STAR*D data consist of four levels. In our analysis, we focus on the 1407 eligible patients who received treatments at Level 2. In particular, at Level 2, patients were asked to indicate their preference of either switching to one of the 4 different treatments, i.e., bupropion (BUP), cognitive therapy (CT), sertraline (SER), and venlafaxine (VEN), or augmenting their existing CIT with 3 options, i.e., CIT+BUP, CIT+buspirone (BUS), and CIT+CT. If a patient indicated no preference, then he/she was assigned to any of the above 7 treatments. To encourage the active collaboration and shared decision-making with patients, we consider the patients’ preference as part of the intervenable treatment options and assume the future patients’ preference can be intervened when recommending treatments. Hence, we have a total of 3+4+7=14 treatment options and these preference-related treatments are often called patient-centered medications in the literature (Robinson et al., 2008). Figure 3 demonstrates the distribution of the observation numbers for the 14 patient-centered treatments. Due to the relatively large treatment space and the unbalanced structure of propensities of treatments, it can be seen that only a few observations were obtained for many treatment options, especially for the treatments in the“no-preference” (Nop-) group.

We apply four methods (SL, PLS, AD, GROWL) discussed in Section 4 to estimate the optimal treatment rules among the 14 treatment options for patients. Specifically, the reward R in our study is calculated by the reduction of QIDS score from the start to the end of Level 2. Hence, a higher value of R is preferred. Feature variables X include QIDS score at the start of Level 2, reduction of QIDS score during Level 1, and other demographic variables such as gender, race, age, education level, employment status, and marital status, etc. The estimated propensities $\hat{p}(A|X)$ are obtained from fitting the multinomial logistic regression model. For PLS, we use terms $\left(1,X,X^{2},A,XA,X^{2}A\right)$ to fit the $L_{1}$ penalized linear regression to estimate conditional treatment effects; For AD, polynomial terms are also included as the basis set of decision functions; For GROWL, we implement the Gaussian kernel for decision functions. Comparisons of all these methods were based on 200 repetitions of three-fold cross-validation, where two folds are used to train the model. For our proposed GROWL, we follow equation (14) discussed in Section 2.4 to determine the number of groups with training data. We evaluate the four methods on the remaining one fold of testing data based on the empirical value function $E_{n}[RI[\hat{D}(X)=A]/\hat{p}(A|X)]/E_{n}[I[\overset{ˆ}{D}(X)=A]/\hat{p}(A|X)]$, where $E_{n}$ denotes the empirical average of testing data.

**Figure 4:**
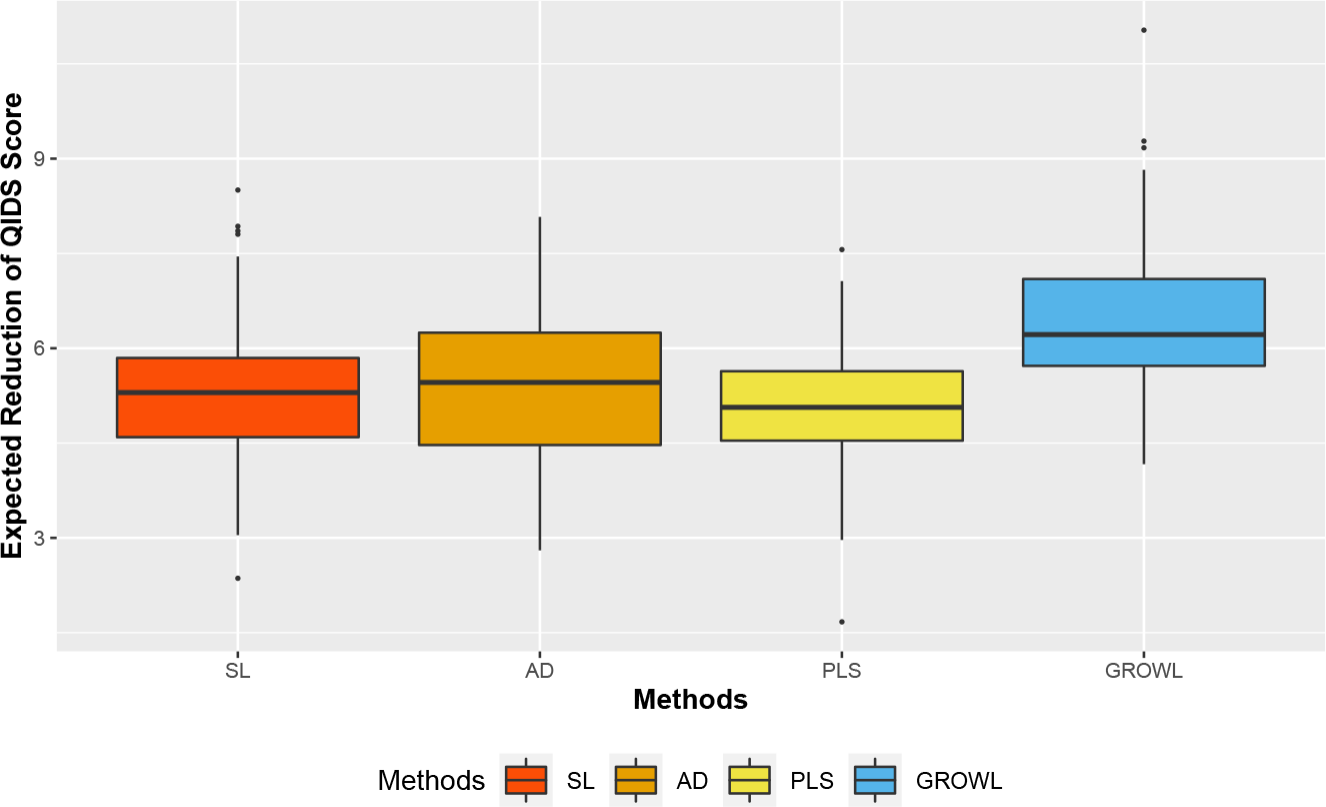
Boxplots of Expected Reduction of QIDS score during Level 2 for patients in testing data based on 200 repetitions for the STAR*D study (higher value is better).

**Figure 5:**
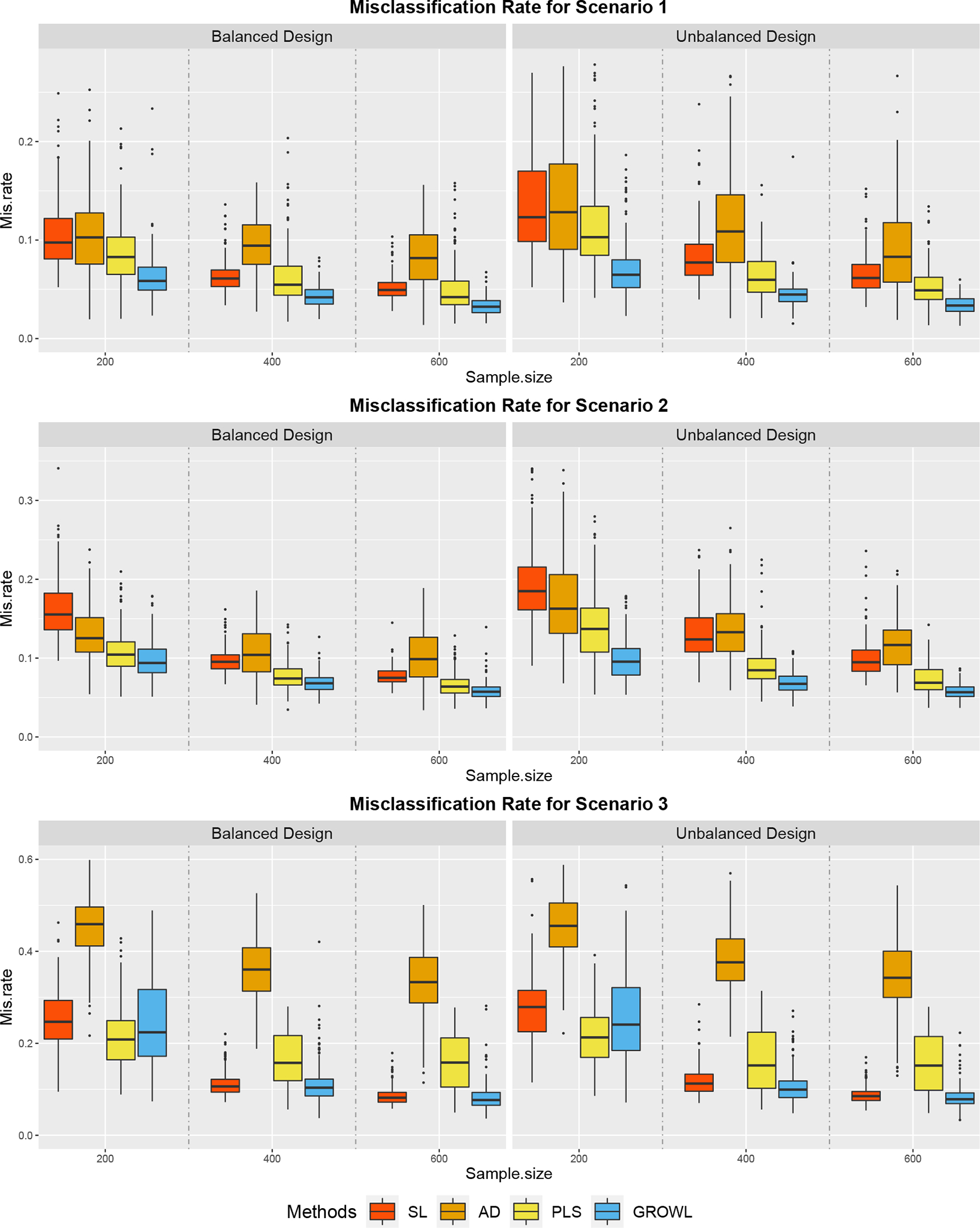
Boxplots of Misclassificaiton Rate evaluated on the independent test data under the **homogeneous** settings.

The testing results are shown in Figure 4. The means of expected reduction of QIDS score during Level 2 by using GROWL is 6.44, which outperform the mean value of SL (5.28), AD (5.32) and PLS (5.04). Thus, compared with methods without considering the treatment partition, GROWL substantially improves the performance of the optimal ITR estimation. The estimated group numbers are 5 or 6 for most of the repetitions. Among the 200 estimated partitions of the treatment space, the patient-centered treatments containing SER, CIT+BUP, CIT+BUS, and CIT+CT are often combined within one group and the treatments containing BUP, CT and VEN are integrated with another group with high frequency. It is interesting to point out that, the treatments SER, CIT+BUP, CIT+BUS, and CIT+CT are often considered as one class of treatments including Selective Serotonin Reuptake Inhibitors (SSRI) while the treatments BUP, CT and VEN are non-SSRI treatments because the treatments within the same group have similar treatment effects (Liu et al., 2018; Pan and Zhao, 2021). In addition, the patient-centered treatments with the same patients’ preference are often clustered into the same group. With the overall dataset, we implement the GROWL with the Gaussian kernel and obtain the final estimated group structure with 5 treatment groups:

$$\begin{array}{l} {\hat{𝒢}}_{0}=\{\{\;\text{Swi-BUP},\;\text{Swi-VEN},\;\text{Nop-VEN}\;\},\{\;\text{Swi-SER}\;\},\\ \;\{\;\text{Aug-CIT+BUP},\;\text{Aug-CIT+BUS},\;\text{Aug-CIT}\;+CT\},\\ \;\{\;\text{Swi-CT},\;\text{Nop-CIT+CT},\;\text{Nop-CT},\;\text{Nop-VEN}\;\},\\ \;\{\;\text{Nop-CIT+BUP},\;\text{Nop-CIT+BUS},\;\text{Nop-SER}\;\}\}. \end{array}$$

It can be seen that these patient-centered treatments with the same preference work similarly within the SSRI groups and the non-SSRI groups respectively.

To better interpret the decision rule and examine the effects of the feature variables, we implement our GROWL with linear kernel based on $\hat{{𝒢}_{0}}$. For the STAR*D dataset, the mean of expected reduction of QIDS score for GROWL with linear kernel is 6.11, demonstrating that GROWL with the linear kernel still outperforms other methods. Note that we have 13 feature variables (including the intercept) and 5 treatment groups. Therefore, we obtain a 4×13 estimated coefficient matrix $\hat{B}$ for the linear decision function. The k-th column of Table 3 in Appendix D demonstrates ${\hat{B}}^{T}W_{k}$ for the k-th treatment group where k=1,2,…,5. We can see that nearly all feature variables play an important role in the estimated optimal ITR. In particular, for the important biomarker, the QIDS score, the patients with higher QIDS score reduction during Level 1 are suggested with augmenting the current CIT treatment implemented at Level 1, while patients with low QIDS reduction are recommended with switching to other treatments at Level 2.

## 6. Discussion

In this article, we propose a new method called GROWL to cluster treatment options and at the same time, estimate the optimal group-structured ITR within one RAMSVM-based objective function. Other comparison methods estimate the ITR under the individual treatment domain while GROWL focuses on the group treatment domain. When homogeneous or nearly-homogeneous treatment group structure is satisfied for the treatments, GROWL is able to find the expected partition with high accuracy and the superior performance of GROWL is demonstrated in our numerical studies. Under heterogeneous settings when θ is small shown in Section 4.2, our method tends to sacrifice the benefit while reduce the variability significantly. In this case, our method is still superior to other methods when the sample size is small. Another advantage of GROWL is that it combines both supervised and unsupervised learning through one single optimization.

From a broad perspective, our method is not limited to ITR problems. It can be viewed as a multicategory classification technique. In particular, consider using observed data $\left(x_{1},y_{1}\right),\ldots ,\left(x_{n},y_{n}\right)$ to classify the covariate x∈𝒳 as a specific class y in a large class space 𝒴. Due to the large number of labels, insufficient data are observed for some specific labels. Consequently, standard classification methods can become ineffective. On the other hand, the conditional probability Pr[Y=y∣X] may employ possible similar patterns for some classes y∈𝒴. Then our method tends to estimate this pattern with group-based structure to reduce the dimension of the classification label space and classify the observations in the group domain. Although our paper mainly focuses on estimating the optimal ITR in the decision making framework, the essence is similar because the conditional treatment effects E[R∣A=a,X] play a similar role as Pr[Y=y∣X] in classification problems.

Several possible extensions can be explored for future studies. First, most scenarios considered in the paper is that the group structure of the treatment effects is independent with the marginal distribution of feature variables X. However, consider the case that homogeneous group structure is completely different with different values of x∈𝒳, the optimal partition in (7) tends to sacrifice some subgroups of individuals. Consequently, more value would be lost because the optimal partition is obtained via averaging the marginal distribution of X. For these more complex scenarios, it will be interesting to estimate different partitions targeting subgroups of individuals. Secondly, our method can be extended to learn group structures for multi-stage Dynamic Treatment Regimes (DTR) (Murphy, 2003; Zhao et al., 2015). This can be an interesting direction for future research.

## Figures and Tables

**Table 1:** Results for Ratio of finding the optimal partition 5° and square root of MSE of Empirical Value Function and Misclassification Rate evaluated on the independent test data under the homogeneous settings. The best values are in bold.

	*n* = 200			*n* = 400			*n* = 600		
	Ratio(%)	Value	Misclassification	Ratio(%) Value	Misclassification	Ratio(%)	Value	Misclassification
**Balanced Design**									
Scenario **1**									
SL	—	0.109	0.112	—	0.046	0.069	—	0.028	0.052
AD	—	0.132	0.126	—	0.056	0.098	—	0.044	0.086
PLS	—	0.070	0.092	—	0.041	0.065	—	0.030	0.057
GROWL	97.0	**0.046**	**0.068**	99.5	**0.014**	**0.044**	100	**0.008**	**0.034**
Scenario **2**									
SL	—	0.181	0.167	—	0.068	0.097	—	0.052	0.078
AD	—	0.106	0.133	—	0.073	0.111	—	0.069	0.107
PLS	—	0.086	0.112	—	0.047	0.079	—	0.041	0.069
GROWL	92.5	**0.079**	**0.101**	98.5	**0.036**	**0.069**	99.0	**0.027**	**0.060**
Scenario **3**									
SL	—	0.567	0.260	—	0.169	0.154	—	0.119	0.108
AD	—	1.310	0.463	—	0.752	0.369	—	0.565	0.342
PLS	—	**0.401**	**0.226**	—	0.199	0.177	—	0.191	0.170
GROWL	67.5	0.511	0.269	92.0	**0.125**	**0.119**	97.5	**0.080**	**0.089**
**Unbalanced Design**									
Scenario **1**									
SL	—	0.258	0.164	—	0.073	0.088	—	0.046	0.068
AD	—	0.416	0.216	—	0.270	0.145	—	0.075	0.101
PLS	—	0.146	0.129	—	0.045	0.067	—	0.032	0.058
GROWL	91.5	**0.085**	**0.078**	99.0	**0.022**	**0.047**	99.0	**0.009**	**0.035**
Scenario **2**									
SL	—	0.255	0.199	—	0.127	0.139	—	0.082	0.102
AD	—	0.246	0.197	—	0.123	0.138	—	0.089	0.120
PLS	—	0.166	0.148	—	0.072	0.093	—	0.047	0.075
GROWL	83.5	**0.080**	**0.103**	97.5	**0.037**	**0.070**	98.0	**0.029**	**0.061**
Scenario **3**									
SL	—	0.675	0.288	—	0.183	0.162	—	0.121	0.112
AD	—	1.484	0.481	—	0.814	0.386	—	0.664	0.355
PLS	—	**0.423**	**0.236**	—	0.231	0.184	—	0.198	0.174
GROWL	56.0	0.570	0.281	83.5	**0.132**	**0.124**	98.0	**0.083**	**0.090**

**Table 2:** Results for square root of MSE of Empirical Value Function evaluated on independent test data under the nonhomogeneous settings and unbalanced designs. The best values are in bold.

	*n* = 200	*n* = 400	*n* = 600
*θ* = 40			
SL	0.265	0.136	0.091
AD	0.424	0.174	0.132
PLS	0.212	0.102	0.076
GROWL	**0.119**	**0.069**	**0.058**
*θ* = 20			
SL	0.266	0.137	0.123
AD	0.480	0.223	0.167
PLS	0.245	0.100	0.084
GROWL	**0.141**	**0.091**	**0.079**
*θ* = 10			
SL	0.306	0.178	0.151
AD	0.546	0.298	0.294
PLS	0.214	**0.112**	**0.091**
GROWL	**0.206**	0.154	0.146
*θ* = 5			
SL	0.339	0.208	0.165
AD	0.664	0.454	0.442
PLS	**0.233**	**0.125**	**0.092**
GROWL	0.315	0.293	0.282

**Figure 6:**
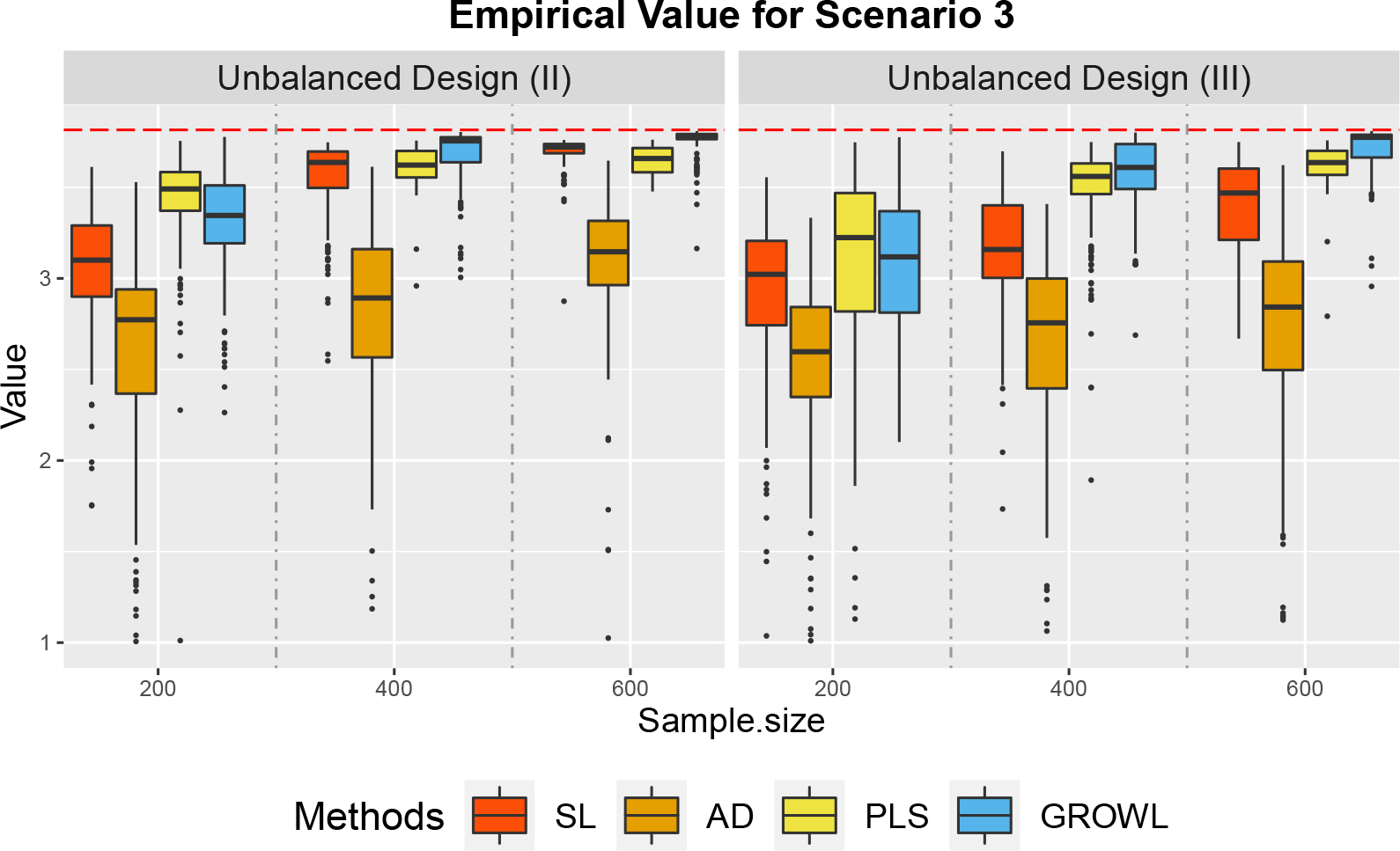
Boxplots of Empirical Value Function under more **unbalanced** designs for the homogeneous case.

**Figure 7:**
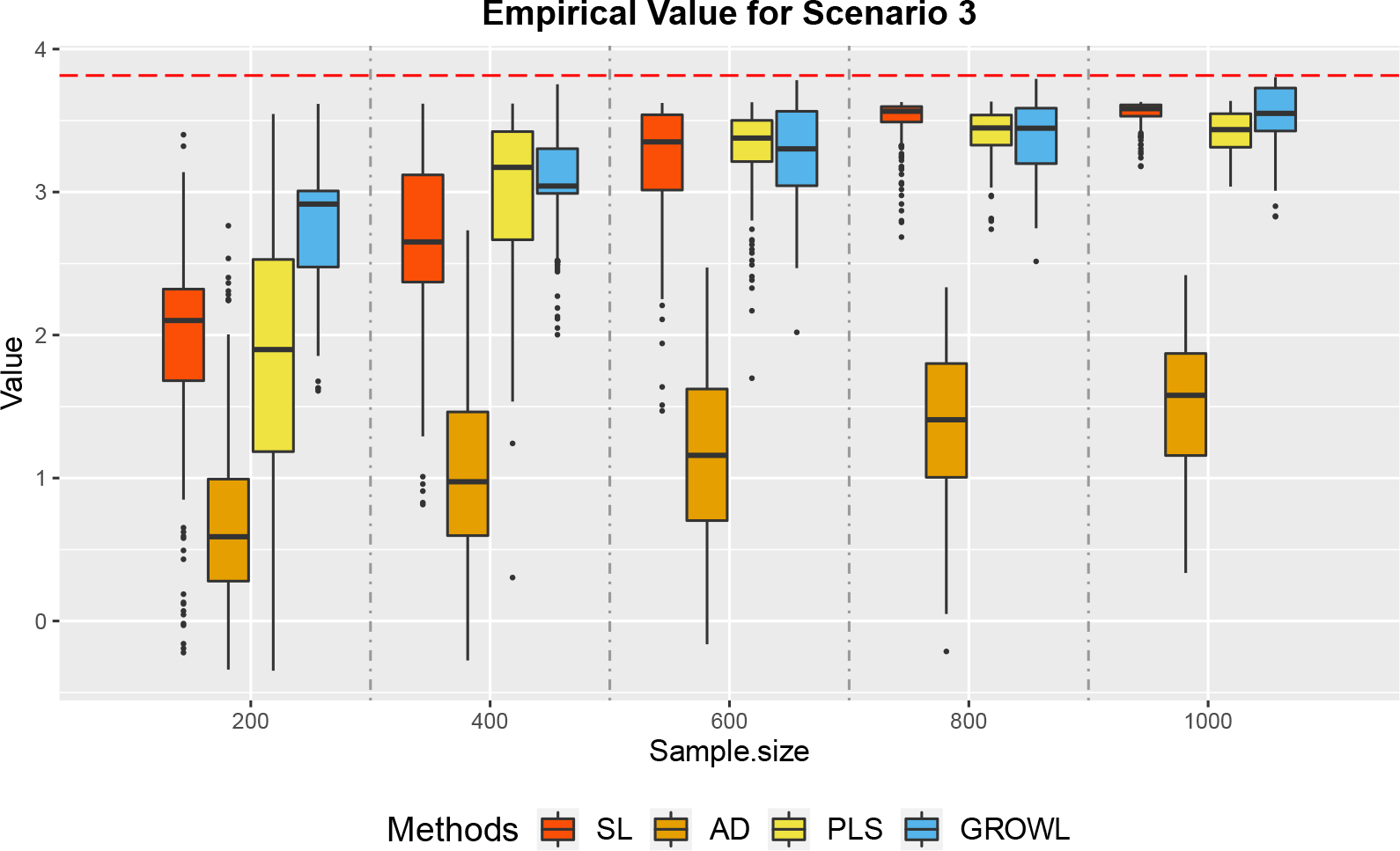
Boxplots of Empirical Value Function under **extreme** designs for the homogeneous case.

**Figure 8:**
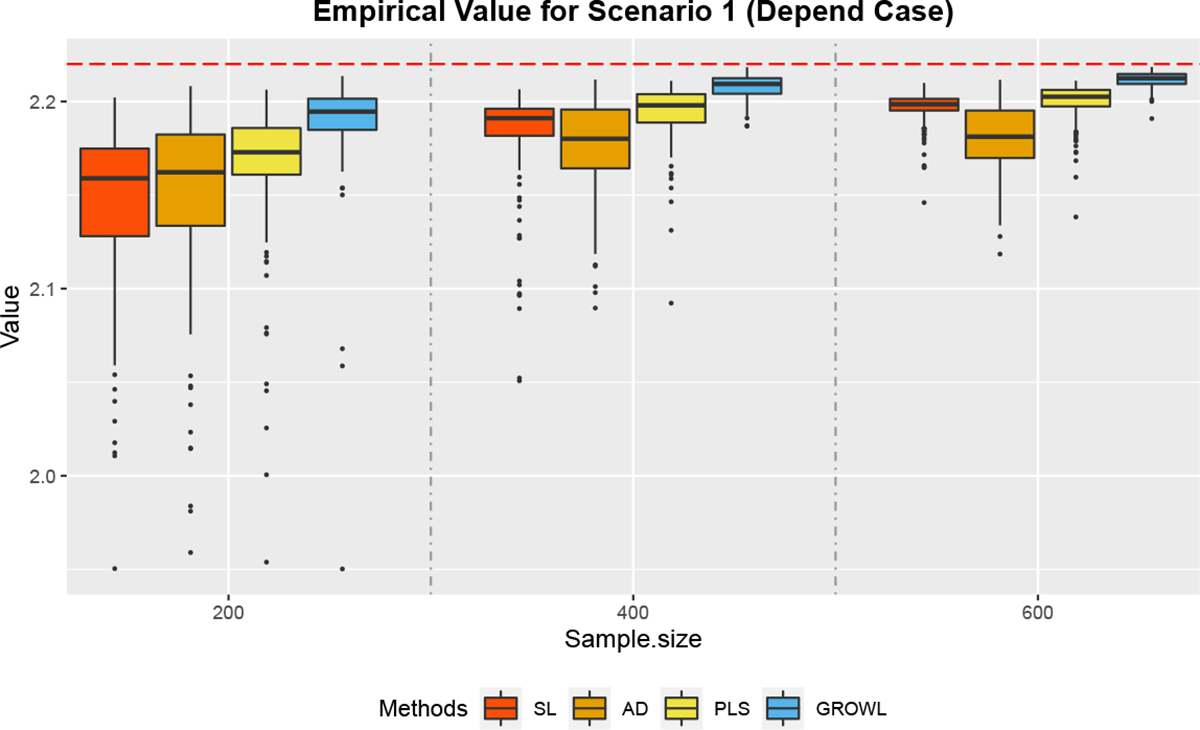
Boxplots of Empirical Value Function under the **dependent** design for the homogeneous case.

**Figure 9:**
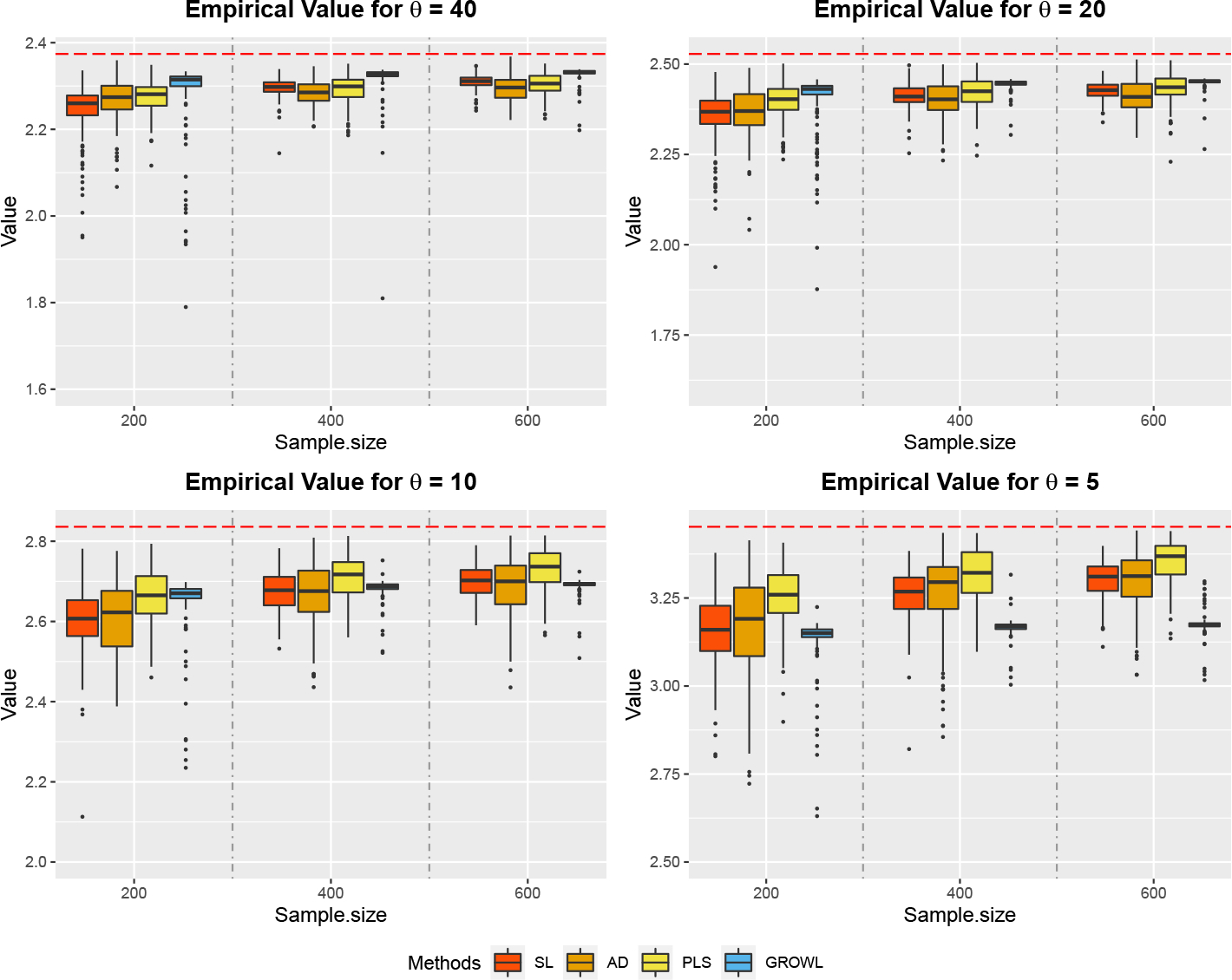
Boxplots of Empirical Value Function under the **dependent** design for the non-homogeneous case.

## Appendix

The appendix contains detailed proofs of the main results, additional figures and tables, and more implementation details. In Appendix A, we introduce some useful lemmas to prove the theorems in the main paper. In Appendix B, we give the detailed proofs of theorems in the main paper. In Appendix C, we prove the lemmas in the main paper and the lemmas in Appendix A. We also provide proofs of equation (10) and how the dual problems (11) and (12) are derived. Additional results of more simulations and real data analysis are given in Appendix D. Finally, implementation details are summarized in Appendix E.

## Appendix A. Useful Lemmas

### Lemma 8 (Bound of excess risk for any fixed δ)

*For any partition* δ, *any measurable function* $f:𝒳\to R^{K_{n}-1}$ *such that* $\left\langle W_{k},f(x)\right\rangle \in \left[-1,K_{n}-1\right]$ *holds for* ∀x∈𝒳 *and* $\forall k\in \left[K_{n}\right]$, *we have*

$$ℛ(\delta ,f)-ℛ(\delta ,f^{\delta })⩽{ℛ}_{\phi }(\delta ,f)-{ℛ}_{\phi }(\delta ,f^{\delta }).$$

Note that Lemma 8 generalizes the excess risk bound in Theorem 3.2 in Zhao et al. (2012) from the binary setting to the multi-class setting for any fixed δ.

### Lemma 9 (Bound for approximation error)

*Fix a partition* δ. *Suppose the generalized geometric noise assumption holds for* δ *with noise exponent* 0<q<∞ *and constant* U. *Set* $\sigma_{n}=\lambda_{n}^{-1/(q+1)d}$. *Then for the Gaussian kernel* κ, *there is a constant* c>0 *depending only on the dimension* d, *the geometric noise exponent* q *and constant* U, *such that for all* $\lambda_{n}>0$, *we have*

$${𝒜}_{\sigma_{n}}^{\delta }\left(\lambda_{n}\right)⩽cK_{n}^{2}\lambda_{n}^{q/(q+1)}.$$

### Lemma 10 (Finite sample regret bound for optimal $\delta^{*}$)

*Suppose the generalized geometric noise assumption holds for an optimal partition* $\delta^{*}\in \Delta^{*}$ *with exponent* 0<q<∞ *and constant* U. *Assume* $|\frac{R}{p\left(\delta^{*}(A)|X\right)}|⩽Z_{n}$. *Then for the Gaussian kernel* κ *and any* θ>0,0<v<2, *there exists a constant* C (*depending on* θ,v,d), *such that for all* τ⩾1 *and* $\sigma_{n}=\lambda_{n}^{-1/(q+1)d}$,

$${Pr}^{*}\left(\tilde{ℛ}(\delta^{*},{\overset{ˆ}{f}}_{n}^{\delta^{*}})⩽{\tilde{ℛ}}^{*}+\epsilon \right)⩾1-e^{-\tau },$$

*where* ${Pr}^{*}$ *denotes the outer probability for possibly nonmeasurable sets, and*

$$\epsilon =CK_{n}Z_{n}\left[{\left(\lambda_{n}\right)}^{-\frac{2}{2+v}+\frac{\left(2-v)(1+\theta \right)}{\left(2+v)(1+q\right)}}n^{-\frac{2}{2+v}}+\frac{\tau }{n\lambda_{n}}+\lambda_{n}^{\frac{q}{q+1}}\right].$$

Note that Lemma 10 is a generalization of Theorem 3.4 in Zhao et al. (2012) to deal with the multiclass ITR problem under the $L_{\phi }$-based loss.

## Appendix B. Proofs of Theorems

### Proof of Theorem 4:

Based on the definition of $L_{\phi }$ loss, ${ℛ}_{\phi }(\delta ,f)$ equals to

$$\begin{array}{l} E_{X,A}\left[\frac{E[R|A,\;X]}{p(\delta (A)|X)}\times [(1-\gamma )\sum_{j\neq \delta (A)}\;{\left(1+\left\langle W_{j},f(X)\right\rangle \right)}^{+}+\gamma {\left(K_{n}-1-\left\langle W_{\delta (A)},f(X)\right\rangle \right)}^{+}]\right]\\ =E_{X}\left[\sum_{k=1}^{K_{n}}\;\sum_{a:\delta (a)=k}\;\frac{E[R|A\;=\;a,\;X]p(a|X)}{p(\delta (A)|X)}\times [(1-\gamma )\sum_{j\neq k}\;{\left(1+\left\langle W_{j},f(X)\right\rangle \right)}^{+}+\gamma {\left(K_{n}-1-\left\langle W_{k},f(X)\right\rangle \right)}^{+}]\right]\\ =E_{X}\left[\sum_{k=1}^{K_{n}}\;E[R|A\in G_{k}^{\delta },X][(1-\gamma )\sum_{j\neq k}\;{\left(1+\left\langle W_{j},f(X)\right\rangle \right)}^{+}+\gamma {\left(K_{n}-1-\left\langle W_{k},f(X)\right\rangle \right)}^{+}\right]. \end{array}$$

For each fixed δ and fixed x∈𝒳, denote $g^{\delta }(x)=arg\;{max}_{k\in \left[K_{n}\right]}\;E[R|A\in G_{k}^{\delta },X=x]$. Then based on the proof of Theorem 1 in Zhang et al. (2016), when γ∈[0,1/2], we have $\langle W_{g^{\delta }(x)},f_{\phi }^{\delta }(x)\rangle =K_{n}-1$ and $\langle W_{j},f_{\phi }^{\delta }(x)\rangle =-1$ for $j\neq g^{\delta }(x)$. Hence, classical Fisher Consistency holds for group-based decision rule for each fixed δ. By plugging $f_{\phi }^{\delta }$ into above equation, we have

$$\begin{array}{l} \underset{f:𝒳\to R^{K_{n}-1}}{inf}\;{ℛ}_{\phi }(\delta ,f)\;={ℛ}_{\phi }(\delta ,f_{\phi }^{\delta })\\ \;=E_{X}\left[\sum_{k\neq g^{\delta }(X)}^{K_{n}}\;\;E[R|A\in G_{k}^{\delta },X]\left[(1-\gamma )K_{n}+\gamma K_{n}\right]\right]\\ \;=E_{X}\left[K_{n}\sum_{k=1}^{K_{n}}\;\;E[R|A\in G_{k}^{\delta },X]-K_{n}\underset{k\in \left[K_{n}\right]}{max}\;E[R|A\in G_{k}^{\delta },X]\right]\\ \;=E\left[K_{n}\frac{R}{p(\delta (A)|X)}\right]-K_{n}E_{X}\left[\underset{k\in \left[K_{n}\right]}{max}\;E[R|A\in G_{k}^{\delta },X]\right]. \end{array}$$

Hence, we have

$$\begin{array}{l} \delta_{\phi }^{*}\;\in \underset{\delta }{arg\;min}\left\{{\tilde{ℛ}}_{\phi }(\delta ,f_{\phi }^{\delta })={ℛ}_{\phi }(\delta ,f_{\phi }^{\delta })-K_{n}E\left[\frac{R}{p(\delta (A)|X)}\right]\right\}\\ \;=\underset{\delta }{arg\;max}\;E_{X}\left[\underset{k\in \left[K_{n}\right]}{max}\;E[R|A\in G_{k}^{\delta },X]\right]=\Delta^{*}. \end{array}$$

Then $arg\;{max}_{k\in \left[K_{n}\right]}\;\langle W_{k},f_{\phi }^{*}(x)\rangle =arg\;{max}_{k\in \left[K_{n}\right]}\;E(R|A\in G_{k}^{\delta^{*}},X=x)$ follows straightforward.

### Proof of Theorem 5:

To prove the excess risk bound, we notice the following decomposition:

$$\begin{array}{l} \tilde{ℛ}(\delta ,f)-{\tilde{ℛ}}^{*}\;=\left(\tilde{ℛ}(\delta ,f)-\tilde{ℛ}(\delta ,f^{\delta })\right)+\left(\tilde{ℛ}(\delta ,f^{\delta })-\tilde{ℛ}(\delta^{*},f^{*})\right)\\ \;=\left(ℛ(\delta ,f)-ℛ(\delta ,f^{\delta })\right)+\left(\tilde{ℛ}(\delta ,f^{\delta })-\tilde{ℛ}(\delta^{*},f^{*})\right)\\ \;⩽\left({ℛ}_{\phi }(\delta ,f)-{ℛ}_{\phi }(\delta ,f^{\delta })\right)+\left(\tilde{ℛ}(\delta ,f^{\delta })-\tilde{ℛ}(\delta^{*},f^{*})\right)\\ \;⩽\left({ℛ}_{\phi }(\delta ,f)-{ℛ}_{\phi }(\delta ,f^{\delta })\right)+\left({\tilde{ℛ}}_{\phi }(\delta ,f^{\delta })-{\tilde{ℛ}}_{\phi }^{*}\right)\\ \;={\tilde{ℛ}}_{\phi }(\delta ,f)-{\tilde{ℛ}}_{\phi }^{*}. \end{array}$$

The first inequality follows from Lemma 8. For the second inequality, notice that

$$\tilde{ℛ}(\delta ,f^{\delta })-\tilde{ℛ}(\delta^{*},f^{*})⩽K_{n}\left(\tilde{ℛ}(\delta ,f^{\delta })-\tilde{ℛ}(\delta^{*},f^{*})\right)={\tilde{ℛ}}_{\phi }(\delta ,f^{\delta })-{\tilde{ℛ}}_{\phi }^{*}.$$

Then the proof is complete.

### Proof of Theorem 6:

For each partition δ, take the following notations. Define the random variable $S^{\delta }=\frac{R}{p(\delta (A)|X)}$. Denote $V_{\phi }^{\delta }(f)=S^{\delta }L_{\phi }(\delta ,f)$ as the weighted loss. Similarly, under δ, let $P^{\delta }$ be the population measure of (X,δ(A),R/p(δ(A)∣X)) and $P_{n}^{\delta }$ be the associated empirical measure. Recall that $\Psi_{n}=\frac{1}{2}{min}_{\delta \notin \Delta^{*}}\;{𝒱}_{1}^{*}(\delta^{*})-{𝒱}_{1}^{*}(\delta )>0$. Then, for any $\delta \notin \Delta^{*}$, we have ${inf}_{f}\;{\tilde{ℛ}}_{\phi }(\delta ,f)-{\tilde{ℛ}}_{\phi }^{*}>\Psi_{n}$. To prove part (I) in this theorem, we need to control the probability of event $\left\{{\hat{\delta }}_{n}\notin \Delta^{*}\right\}$. Take an optimal $\delta^{*}\in \Delta^{*}$. Note that based on the definition of ${\hat{\delta }}_{n}$, the following two events are equivalent:

$$\left\{{\hat{\delta }}_{n}\in {\text{Δ}}^{\text{*}}\right\}\Leftrightarrow \underset{\delta \notin {\text{Δ}}^{\text{*}}}{\cap }\left\{{\mathbb{P}}_{n}^{\delta^{\text{*}}}\left(V_{\phi }^{\delta \text{*}}\left({\hat{f}}_{n}\right)\right)+\lambda_{n}{\left\|{\hat{f}}_{n}\right\|}_{{ℱ}_{n}}^{2}-K_{n}{\mathbb{P}}_{n}^{\delta^{\text{*}}}\left(S^{\delta^{\text{*}}}\right)⩽{\mathbb{P}}_{n}^{\delta }\left(V_{\phi }^{\delta }\left({\hat{f}}_{n}\right)\right)+\lambda_{n}{\left\|{\hat{f}}_{n}\right\|}_{{ℱ}_{n}}^{2}-K_{n}{\mathbb{P}}_{n}^{\delta }\left(S^{\delta }\right)\right\}.$$

Hence, $Pr\left[{\hat{\delta }}_{n}\in \Delta^{*}\right]$

$$\;=1-Pr\left[\underset{\delta \notin \Delta^{*}}{⋃}\;\;\left\{P_{n}^{\delta^{*}}(V_{\phi }^{\delta^{*}}({\overset{ˆ}{f}}_{n}))+\lambda_{n}{∥{\overset{ˆ}{f}}_{n}∥}_{{ℱ}_{n}}^{2}-K_{n}P_{n}^{\delta^{*}}(S^{\delta^{*}})>P_{n}^{\delta }(V_{\phi }^{\delta }({\overset{ˆ}{f}}_{n}))+\lambda_{n}{∥{\overset{ˆ}{f}}_{n}∥}_{{ℱ}_{n}}^{2}-K_{n}P_{n}^{\delta }(S^{\delta })\right\}\right]\;\;⩾1-\sum_{\delta \notin \Delta^{*}}\;\;Pr\left[P_{n}^{\delta^{*}}(V_{\phi }^{\delta^{*}}({\overset{ˆ}{f}}_{n}))+\lambda_{n}{∥{\overset{ˆ}{f}}_{n}∥}_{{ℱ}_{n}}^{2}-K_{n}P_{n}^{\delta^{*}}(S^{\delta^{*}})>P_{n}^{\delta }(V_{\phi }^{\delta }({\overset{ˆ}{f}}_{n}))+\lambda_{n}{∥{\overset{ˆ}{f}}_{n}∥}_{{ℱ}_{n}}^{2}-K_{n}P_{n}^{\delta }(S^{\delta })\right]\;\;=1-\sum_{\delta \notin \Delta *}\;\;\left(1-Pr\left[P_{n}^{\delta^{*}}(V_{\phi }^{\delta^{*}}({\overset{ˆ}{f}}_{n}))+\lambda_{n}{∥{\overset{ˆ}{f}}_{n}∥}_{{ℱ}_{n}}^{2}-K_{n}P_{n}^{\delta^{*}}(S^{\delta^{*}})⩽P_{n}^{\delta }(V_{\phi }^{\delta }({\overset{ˆ}{f}}_{n}))+\lambda_{n}{∥{\overset{ˆ}{f}}_{n}∥}_{{ℱ}_{n}}^{2}-K_{n}P_{n}^{\delta }(S^{\delta })\right]\right)$$

After rearranging both sides, we can only control the following probability for each fixed $\delta \notin \Delta^{*}$ in order to lower bound $\left[{\hat{\delta }}_{n}\in \Delta^{*}\right]$:

$$\begin{array}{l} Pr\left[P_{n}^{\delta^{*}}(V_{\phi }^{\delta^{*}}({\overset{ˆ}{f}}_{n}))-P_{n}^{\delta }(V_{\phi }^{\delta }({\overset{ˆ}{f}}_{n}))-K_{n}(P_{n}^{\delta^{*}}(S^{\delta^{*}})-P_{n}^{\delta }(S^{\delta }))⩽0\right]\\ =Pr\left[P_{n}^{\delta^{*}}(V_{\phi }^{\delta^{*}}({\overset{ˆ}{f}}_{n}))-P_{n}^{\delta }(V_{\phi }^{\delta }({\overset{ˆ}{f}}_{n}))-K_{n}(P_{n}^{\delta^{*}}(S^{\delta^{*}})-P_{n}^{\delta }(S^{\delta }))-({\tilde{ℛ}}_{\phi }^{*}-{\tilde{ℛ}}_{\phi }(\delta ,f^{\delta }))⩽{\tilde{ℛ}}_{\phi }(\delta ,f^{\delta })-{\tilde{ℛ}}_{\phi }^{*}\right]\\ ⩾Pr\left[P_{n}^{\delta^{*}}(V_{\phi }^{\delta^{*}}({\overset{ˆ}{f}}_{n}))-P_{n}^{\delta }(V_{\phi }^{\delta }({\overset{ˆ}{f}}_{n}))-K_{n}(P_{n}^{\delta^{*}}(S^{\delta^{*}})-P_{n}^{\delta }(S^{\delta }))-({\tilde{ℛ}}_{\phi }^{*}-{\tilde{ℛ}}_{\phi }(\delta ,f^{\delta }))⩽\Psi_{n}\right]\\ =Pr\left[I+II+III-IV⩽\Psi_{n}\right]\\ ⩾Pr\left[I+II+III⩽\Psi_{n}\right], \end{array}$$

where

$$\begin{array}{l} I=(P_{n}^{\delta^{*}}-P^{\delta^{*}})(V_{\phi }^{\delta^{*}}({\overset{ˆ}{f}}_{n}))-(P_{n}^{\delta }-P^{\delta })(V_{\phi }^{\delta }({\overset{ˆ}{f}}_{n})),\\ II=K_{n}(P^{\delta^{*}}-P_{n}^{\delta^{*}})(S^{\delta^{*}})-K_{n}(P^{\delta }-P_{n}^{\delta })(S^{\delta }),\\ III=P^{\delta^{*}}(V_{\phi }^{\delta^{*}}({\hat{f}}_{n}-f^{\delta^{*}})),\\ IV=P^{\delta }(V_{\phi }^{\delta }({\hat{f}}_{n}-f^{\delta })). \end{array}$$

Note that the last inequality above is due to IV⩾0. Here, after removing IV, the advantage is that we only need the generalized geometric noise condition holds for $\delta^{*}$ to bound III following from Lemma 10.

Now, we would bound the above three terms respectively using concentration techniques. First, to bound I, we start with obtaining a bound for ${∥{\overset{ˆ}{f}}_{n}∥}_{{ℱ}_{n}}^{2}$. Since $P_{n}^{{\overset{ˆ}{\delta }}_{n}}(V_{\phi }^{{\hat{\delta }}_{n}}({\overset{ˆ}{f}}_{n})-K_{n}S^{{\overset{ˆ}{\delta }}_{n}})+\lambda_{n}{∥{\overset{ˆ}{f}}_{n}∥}_{{ℱ}_{n}}^{2}⩽P_{n}^{\delta }(V_{\phi }^{\delta }(f)-K_{n}S^{\delta })+\lambda_{n}∥f{∥}_{{ℱ}_{n}}^{2}$ holds for any δ and $f\in {⊗}_{k=1}^{K_{n}-1}{ℋ}_{\kappa }^{k}$, we can select $\delta ={\hat{\delta }}_{n}$ and f=0 to get

$${∥{\hat{f}}_{n}∥}_{{ℱ}_{n}}^{2}⩽\frac{1}{\lambda_{n}}\frac{1}{n}\sum_{i=1}^{n}\;S_{i}^{\delta }L_{\phi }({\hat{\delta }}_{n},0)⩽\frac{2\left(K_{n}\;-\;1\right)}{\lambda_{n}}Z_{n},$$

so that only the constrained class ${ℱ}_{n}=\left\{\sqrt{\lambda_{n}}f\in {⊗}_{k=1}^{K_{n}-1}{ℋ}_{\kappa }^{k}:{∥\sqrt{\lambda_{n}}f∥}_{{ℱ}_{n}}⩽\sqrt{2\left(K_{n}-1\right)Z_{n}}\right\}$ would be considered. Note that RAMSVM loss $L_{\phi }$ is Lipschitz continuous with respect to f with Lipschitz constant $K_{n}Z_{n}$. Then, for each δ, McDiarmid’s inequality (Bartlett and Mendelson, 2002) implies that with probability at least $1-e^{-\tau }$,

$$\underset{f\in {ℱ}_{n}}{sup}\;\left(P_{n}^{\delta }-P^{\delta }\right)V_{\phi }^{\delta }(f)⩽E\left[\underset{f\in {ℱ}_{n}}{sup}\;\left(P_{n}^{\delta }-P^{\delta }\right)V_{\phi }^{\delta }(f)\right]+2K_{n}Z_{n}\sqrt{\frac{2\tau }{n}}.$$

Next we define the Rademacher complexity over ${ℱ}_{n}$ as $R_{n}^{\delta }:=E{sup}_{f\in {ℱ}_{n}}\;P_{n}\left(\varepsilon V_{\phi }^{\delta }(f)\right)$, where ε is the Rademacher variable independent of $\left(X,\delta (a),S^{\delta }\right)$ under $P^{\delta }$. Then by standard symmetrization arguments and Lemma 22 in Bartlett and Mendelson (2002), we have

$$E\left[\underset{f\in {ℱ}_{n}}{sup}\;\left(P_{n}^{\delta }-P^{\delta }\right)V_{\phi }^{\delta }(f)\right]⩽2R_{n}^{\delta }⩽2\sqrt{\frac{2\left(K_{n}\;-\;1\right)Z_{n}}{n\lambda_{n}}}.$$

Similar proof holds for $P^{\delta }-P_{n}^{\delta }$. Therefore, we have $Pr\left[I⩽\epsilon_{1}\right]⩾1-2e^{-\tau }$, where

$$\epsilon_{1}=4\sqrt{\frac{2\left(K_{n}\;-\;1\right)Z_{n}}{n\lambda_{n}}}+4K_{n}Z_{n}\sqrt{\frac{2\tau }{n}}$$

Next we bound the second term II. Note that by assumption, for each $\delta ,\left(K_{n}-1\right)\left|S^{\delta }\right|⩽\left(K_{n}-1\right)Z_{n}$. By Hoeffding inequality (Steinwart and Christmann, 2008), we have

$$Pr\left[II⩽\epsilon_{2}\right]⩾1-e^{-\tau },$$

where

$$\epsilon_{2}=\left(K_{n}-1\right)Z_{n}\sqrt{\frac{\tau }{2n}}.$$

Finally, bounding III with Lemma 10, we have for any θ>0,v∈[0,2], such that for all τ⩾1 and $\sigma_{n}=\lambda_{n}^{-1/(q+1)d}$, we have

$${Pr}^{*}[{ℛ}_{\phi }(\delta^{*},{\hat{f}}_{n}^{\delta^{*}})⩽{ℛ}_{\phi }^{*}+\epsilon_{3}]=Pr\left[III⩽\epsilon_{3}\right]⩾1-e^{-\tau },$$

and

$$\epsilon_{3}=CK_{n}Z_{n}\left[{\left(\lambda_{n}\right)}^{-\frac{2}{2+v}+\frac{\left(2-v)(1+\theta \right)}{\left(2+v)(1+q\right)}}n^{-\frac{2}{2+v}}+\frac{\tau }{n\lambda_{n}}+\lambda_{n}^{\frac{q}{q+1}}\right],$$

where C is a constant depending on v,θ and d.

Therefore, combining the bound for I, II, III, we have

$${Pr}^{*}\left[I+II+III⩽\epsilon_{1}+\epsilon_{2}+\epsilon_{3}\right]⩾1-4e^{-\tau },$$

where $\epsilon_{1},\epsilon_{2}$ and $\epsilon_{3}$ depend on n and go to 0 as n→∞. Note that $\epsilon_{1}=𝒪(K_{n}^{1/2}Z_{n}^{1/2}n^{-1/2}\lambda_{n}^{-1/2}+K_{n}Z_{n}n^{-1/2})$, $\epsilon_{2}=𝒪\left(K_{n}Z_{n}n^{-1/2}\right)$ and $\epsilon_{3}=𝒪\left(K_{n}Z_{n}{\left(\lambda_{n}\right)}^{-\frac{2}{2+v}+\frac{\left(2-v)(1+\theta \right)}{\left(2+v)(1+q\right)}}n^{-\frac{2}{2+v}}+\right.\left.K_{n}Z_{n}{\left(n\lambda_{n}\right)}^{-1}+K_{n}Z_{n}\lambda_{n}^{\frac{q}{q+1}}\right)$. Based on $\Psi_{n}/\left(\epsilon_{1}+\epsilon_{2}+\epsilon_{3}\right)\to \infty$, we can find sufficient larget n such that $\epsilon_{1}+\epsilon_{2}+\epsilon_{3}<\Psi_{n}$, then we have

$${Pr}^{*}[{\hat{\delta }}_{n}\in \Delta^{*}]=1-𝒪\left(\Gamma_{n}\epsilon_{n}\right).$$

To prove (II), for each fixed τ>1,θ>0,0<v<2, consider

$$\begin{array}{l} Pr\left[{\tilde{ℛ}}_{\phi }({\overset{ˆ}{\delta }}_{n},{\hat{f}}_{n})-{\tilde{ℛ}}_{\phi }^{*}⩾2\epsilon_{3}\right]\\ =Pr\left[\left({\tilde{ℛ}}_{\phi }({\hat{\delta }}_{n},{\hat{f}}_{n})-{\tilde{ℛ}}_{\phi }(\delta^{*},{\hat{f}}_{n})\right)+\left({\tilde{ℛ}}_{\phi }(\delta^{*},{\hat{f}}_{n})-{\tilde{ℛ}}_{\phi }^{*}\right)⩾2\epsilon_{3}\right]\\ ⩽Pr\left[{\tilde{ℛ}}_{\phi }({\hat{\delta }}_{n},{\hat{f}}_{n})-{\tilde{ℛ}}_{\phi }(\delta^{*},{\hat{f}}_{n})⩾\epsilon_{3}\right]+Pr\left[{\tilde{ℛ}}_{\phi }(\delta^{*},{\hat{f}}_{n})-{\tilde{ℛ}}_{\phi }^{*}⩾\epsilon_{3}\right]. \end{array}$$

Next we bound the above two terms respectively. For the first term, take n sufficient large such that $\epsilon_{1}+\epsilon_{2}+\epsilon_{3}<\Psi_{n}$, then

$$\begin{array}{l} Pr\left[{\tilde{ℛ}}_{\phi }({\hat{\delta }}_{n},{\overset{ˆ}{f}}_{n})-{\tilde{ℛ}}_{\phi }(\delta^{*},{\overset{ˆ}{f}}_{n})⩾\epsilon_{3}\right]\\ =Pr\left[{\tilde{ℛ}}_{\phi }({\hat{\delta }}_{n},{\hat{f}}_{n})-{\tilde{ℛ}}_{\phi }(\delta^{*},{\hat{f}}_{n})⩾\epsilon_{3}|{\hat{\delta }}_{n}\in \Delta^{*}\right]Pr\left[{\hat{\delta }}_{n}\in \Delta^{*}\right]\\ \;+Pr\left[{\tilde{ℛ}}_{\phi }({\hat{\delta }}_{n},{\overset{ˆ}{f}}_{n})-{\tilde{ℛ}}_{\phi }(\delta^{*},{\hat{f}}_{n})⩾\epsilon_{3}|{\hat{\delta }}_{n}\notin \Delta^{*}\right]Pr\left[{\hat{\delta }}_{n}\notin \Delta^{*}\right]\\ ⩽0\;+Pr\left[{\hat{\delta }}_{n}\notin \Delta^{*}\right]\\ ⩽4e^{-\tau }. \end{array}$$

For the second term, Lemma 10 implies that $Pr\left[{\tilde{ℛ}}_{\phi }(\delta^{*},{\hat{f}}_{n})-{\tilde{ℛ}}_{\phi }^{*}⩾\epsilon_{3}\right]⩽e^{-\tau }$. Combining both parts, when $\epsilon_{1}+\epsilon_{2}+\epsilon_{3}<\Psi_{n}$, we have for all τ⩾1,

$$Pr\left[{\tilde{ℛ}}_{\phi }({\hat{\delta }}_{n},{\hat{f}}_{n})-{\tilde{ℛ}}_{\phi }^{*}⩽𝒪\left(\epsilon_{1}+\epsilon_{2}+\epsilon_{3}\right)\right]⩾1-5e^{-\tau }.$$

Therefore, we have

$$\tilde{ℛ}({\hat{\delta }}_{n},{\hat{f}}_{n})-{\tilde{ℛ}}^{*}={𝒪}_{p}\left(\Gamma_{n}\epsilon_{n}\right).$$

Note that when R is replaced with $R^{*}=(R\;-\;s(X){)}^{+}+\left(K_{n}-1\right)(R\;-\;s(X){)}^{-}$ discussed in Section 2.3, the final convergence rate would also include the rate coming from estimating s(X). More details can be found in Liu et al. (2018).

## Appendix C. Proofs of Lemmas, Equation (10), and Dual Problems (11) and (12)

### Proof of Lemma 2:

For any fixed $\delta :𝒜\to \left[K_{n}\right]$,

$$\begin{array}{l} {𝒱}_{1}^{*}(\delta )\;=\underset{D_{g}:𝒳\to \left[K_{n}\right]}{max}\;E\left[I\left[D_{g}(X)=\delta (A)\right]\frac{R}{p(\delta (A)|X)}\right]\\ \;=\underset{D_{g}:𝒳\to \left[K_{n}\right]}{max}\;E_{X}\left[\sum_{a\in 𝒜}\;\;I\left[D_{g}(X)=\delta (a)\right]\frac{p(a|X)}{p(\delta (a)|X)}E[R|X,A=a]\right]\\ \;=\underset{D_{g}:𝒳\to \left[K_{n}\right]}{max}\;E_{X}\left[\sum_{k=1}^{K_{n}}\;\;I\left[D_{g}(X)=k\right]\sum_{a:\delta (a)=k}\;\;\frac{p(a|X)}{p(\delta (a)|X)}E[R|X,A=a]\right]\\ \;=E_{X}[\underset{k\in \left[K_{n}\right]}{max}\;\sum_{a:\delta (a)=k}\;\;\frac{p(a|X)}{p(\delta (a)|X)}E[R|X,A=a]]\\ \;⩽E_{X}\left[\underset{a\in 𝒜}{max}\;E[R|X,A=a]\right]\\ \;=E_{X}\left[\underset{k\in \left[K_{n}\right]}{max}\;E\left[R|X,A\in G_{k}^{0}\right]\right]={𝒱}_{1}^{*}\left(\delta^{0}\right), \end{array}$$

where the last equation is because of the homogeneous group structure. So, taking supremum over all of the partitions gives ${sup}_{\delta :𝒜\to \left[K_{n}\right]}\;{𝒱}_{1}^{*}(\delta )⩽{𝒱}_{1}^{*}\left(\delta^{0}\right)$. Therefore, based on the definition of $\delta^{*}$, we have $\delta^{*}=\delta^{0}$.

### Proof of Lemma 8:

First, for the excess risk of 0–1 loss,

$$\begin{array}{l} ℛ(\delta ,f)-ℛ(\delta ,f^{\delta })\\ =E\left[\frac{R}{p(\delta (A)|X)}(I[\underset{j}{arg\;max}\left\langle W_{j},f(X)\right\rangle \neq \delta (A)]-I[arg\;\underset{j}{max}\;\langle W_{j},f^{\delta }(X)\rangle \neq \delta (A)])\right]\\ =E\left[\sum_{k=1}^{K_{n}}\;\;E[R|A\in G_{k}^{\delta },X](I[arg\;\underset{j}{max}\langle W_{j},f(X)\rangle \neq \delta (A)]-I[arg\;\underset{j}{max}\;\langle W_{j},f^{\delta }(X)\rangle \neq \delta (A)])\right] \end{array}$$

Then, for the excess risk of RAMSVM loss,

$$\begin{array}{l} {ℛ}_{\phi }(\delta ,f)-{ℛ}_{\phi }(\delta ,f^{\delta })\\ =E\left[\frac{R}{p(\delta (A)|X)}((1-\gamma )\sum_{k\neq \delta (A)}{\left(1+\left\langle W_{k},f(X)\right\rangle \right)}^{+}+\gamma {\left(K_{n}-1-\left\langle W_{\delta (A)},f(X)\right\rangle \right)}^{+}\;)\right]\\ \;-E\left[\frac{R}{p(\delta (A)|X)}((1-\gamma )\sum_{k\neq \delta (A)}\;\;{\left(1+\langle W_{k},f^{\delta }(X)\rangle \right)}^{+}+\gamma {\left(K_{n}-1-\langle W_{\delta (A)},f^{\delta }(X)\rangle \right)}^{+})\right]\\ =E\left[\sum_{k=1}^{K_{n}}\;\;E[R|A\in G_{k}^{\delta },X]((1-\gamma )\sum_{k\neq \delta (A)}\;{\left(1+\left\langle W_{k},f(X)\right\rangle \right)}^{+}+\gamma {\left(K_{n}-1-\left\langle W_{\delta (A)},f(X)\right\rangle \right)}^{+})\right]\\ \;-E\left[\sum_{k=1}^{K_{n}}\;\;E[R|A\in G_{k}^{\delta },X]((1-\gamma )\sum_{k\neq \delta (A)}\;\;{\left(1+\langle W_{k},f^{\delta }(X)\rangle \right)}^{+}+\gamma {\left(K_{n}-1-\langle W_{\delta (A)},f^{\delta }(X)\rangle \right)}^{+})\right]\\ ⩾E\left[\sum_{k=1}^{K_{n}}\;\;E[R|A\in G_{k}^{\delta },X](\left(1-\gamma )\sum_{k\neq \delta (A)}\;\;(1+\langle W_{k},f(X)\rangle )+\gamma (K_{n}-1-\langle W_{\delta (A)},f(X)\rangle \right))\right]\\ \;-E\left[\sum_{k=1}^{K_{n}}\;\;E[R|A\in G_{k}^{\delta },X](\left(1-\gamma )\sum_{k\neq \delta (A)}\;\;(1+\langle W_{k},f^{\delta }(X)\rangle )+\gamma (K_{n}-1-\langle W_{\delta (A)},f^{\delta }(X)\rangle \right))\right]\\ =E\left[\sum_{k=1}^{K_{n}}\;\;E[R|A\in G_{k}^{\delta },X](\left(K_{n}-1-\langle W_{k},f(X)\rangle )-(K_{n}-1-\langle W_{k},f^{\delta }(X)\rangle \right))\right]\\ =E\left[\sum_{k=1}^{K_{n}}\;\;E[R|A\in G_{k}^{\delta },X]\langle W_{k},f^{\delta }(X)-f(X)\rangle \right]. \end{array}$$

The inequality is because, taking out the positive operator would make the first term smaller while leave the second term unchanged due to $\langle W_{k},f^{\delta }(x)\rangle \in \left[-1,K_{n}-1\right]$ for every x∈𝒳. Next, we can only prove, for each x∈𝒳,

(19)
$$\begin{array}{l} E\left[\sum_{k=1}^{K_{n}}\;\;E[R|A\in G_{k}^{\delta },X=x]\langle W_{k},f^{\delta }(x)-f(x)\rangle \right]\\ \;⩾E\left[\sum_{k=1}^{K_{n}}\;\;E[R|A\in G_{k}^{\delta },X=x](I[\underset{j}{\arg max}\langle W_{j},f(x)\rangle \neq \delta (A)]-I[\arg\underset{j}{max}\;\langle W_{j},f^{\delta }(x)\rangle \neq \delta (A)])\right]. \end{array}$$

Suppose $E[R|A\in G_{s}^{\delta },X=x]>E[R|A\in G_{k}^{\delta },X=x]$ for every k≠s. Then based on Fisher Consistency for group-based decision rule, we have $arg\;{max}_{j\in \left[K_{n}\right]}\;\langle W_{j},f^{\delta }(x)\rangle =s$. Suppose $arg\;{max}_{j}\;\left\langle W_{j},f(x)\right\rangle =r$ where r≠s. The right hand side of (19) becomes

$$\begin{array}{l} \;\sum_{k\neq r,s}^{K_{n}}\;\;E[R|A\in G_{k}^{\delta },X=x](1-1)+E[R|A\in G_{r}^{\delta },X=x](0-1)+E[R|A\in G_{s}^{\delta },X=x](1-0)\\ =E[R|A\in G_{s}^{\delta },X=x]-E[R|A\in G_{r}^{\delta },X=x]. \end{array}$$

Since $\sum_{k=1}^{K_{n}}\;W_{k}=0$, the left hand side of (19) equals to

$$\begin{array}{l} \;\sum_{k\neq s}^{K_{n}}\;\;E[R|A\in G_{s}^{\delta },X=x]\langle -W_{k},f^{\delta }(x)-f(x)\rangle +\sum_{k\neq s}^{K_{n}}\;\;E[R|A\in G_{k}^{\delta },X=x]\langle W_{k},f^{\delta }(x)-f(x)\rangle \\ =\;\sum_{k\neq s}^{K_{n}}\;\;\left[E[R|A\in G_{k}^{\delta },X=x]-E[R|A\in G_{s}^{\delta },X=x]\right]\langle W_{k},f^{\delta }(x)-f(x)\rangle \\ =\;\sum_{k\neq r,s}^{K_{n}}\;\;\left[E[R|A\in G_{s}^{\delta },X=x]-E[R|A\in G_{k}^{\delta },X=x]\right]\left(1+⟠W_{k},f(x)\rangle \right)\\ \;+\left[E[R|A\in G_{s}^{\delta },X=x]-E[R|A\in G_{r}^{\delta },X=x]\right]\left(1+\langle W_{r},f(x)\rangle \right). \end{array}$$

The last equation is based on $\langle W_{k},f^{\delta }(x)\rangle =-1$ for each k≠s (Theorem 1 in Zhang et al. (2016)). Then, by the definition of f and the constraint of bound, we have $\left\langle W_{k},f(x)\right\rangle ⩾-1$ for k≠r and $\left\langle W_{r},f(x)\right\rangle ⩾0$. Hence, (19) holds and taking expectation for X in both sides of (19) gives the result.

### Proof of Lemma 9:

Let ${ℋ}_{\kappa }\left(R^{d}\right)$ be the RKHS of the RBF kernel with parameter $\sigma_{n}$. Then based on Steinwart and Scovel (2007), the following linear operator $V_{\sigma_{n}}:{⊗}_{k=1}^{K_{n}-1}L_{2}^{k}\left(R^{d}\right)\to {⊗}_{k=1}^{K_{n}-1}{ℋ}_{\kappa }^{k}\left(R^{d}\right)$ defined by

$$V_{\sigma_{n}}g(x)=\frac{{\left(2\sigma_{n}\right)}^{d/2}}{\pi^{d/4}}\int_{R^{d}}\;e^{-2\sigma_{n}^{2}∥x-y{∥}^{2}}g(y)\;dy,\;g\in {⊗}_{k=1}^{K_{n}-1}L_{2}^{k}\left(R^{d}\right),x\in R^{d},$$

is an isometric isomorphism. Accordingly, for any fixed δ, we obtain

$${𝒜}_{\sigma_{n}}^{\delta }\left(\lambda_{n}\right)⩽\underset{\left\langle V_{\sigma_{n}}g,W_{k}\right\rangle \in \left[-1,K_{n}-1\right],\forall k\in \left[K_{n}\right]}{inf}\;\lambda_{n}∥g{∥}_{L_{2}}^{2}+{ℛ}_{\phi }\left(\delta ,V_{\sigma_{n}}g\right)-{ℛ}_{\phi }(\delta ,f^{\delta }).$$

Using the notation of Lemma 4.1 of Steinwart and Scovel (2007), define $\overset{\prime }{𝒳}=3𝒳$. Let ${\overset{\prime }{𝒳}}_{i}^{\delta }$ be similarly defined as ${𝒳}_{i}^{\delta }$ on the domain of $\overset{\prime }{𝒳}$. We fix a specific measurable function ${\overset{\prime }{f}}^{\delta }$ such that for each $i\in \left[K_{n}\right]$ and $x\in {\overset{\prime }{𝒳}}_{i}^{\delta },{\overset{\prime }{f}}^{\delta }$ satisfies $\langle W_{i},{\overset{\prime }{f}}^{\delta }\left(x\right)\rangle =K_{n}-1$ and $\langle W_{j},{\overset{\prime }{f}}^{\delta }\left(x\right)\rangle =-1$ for j≠i. For $x\in \overset{\prime }{𝒳}$, it can be checked that the above property is equivalent with $f^{\delta }\left(x\right)=\left(K_{n}-1\right)\sum_{i=1}^{K_{n}}W_{i}I\left[x\in {\overset{\prime }{𝒳}}_{i}^{\delta }\right]$. Besides $\overset{\prime }{𝒳}$, define ${\overset{\prime }{f}}^{\delta }\left(x\right)=0$. By similar approach of Lemma 4.1 in Steinwart and Scovel (2007), we can make sure the ball $B\left(x,{\text{Δ}}^{\delta }\left(x\right)\right)\subset {\overset{\prime }{𝒳}}_{i}^{\delta }$ for every $x\in {𝒳}_{i}^{\delta }\left(i\in \left[K_{n}\right]\right)$ on this enlarged support for 𝒳. Choose $g={\left(\sigma_{n}^{2}/\pi \right)}^{d/4}{\overset{\prime }{f}}^{\delta }$ and note that ${∥W_{i}∥}_{2}=1$, we immediately obtain the following with Jensen inequality and assumption of bounded volume of 𝒳,

$$∥g{∥}_{L_{2}}^{2}⩽\left(K_{n}-1\right){\left(\frac{81\sigma_{n}^{2}}{\pi }\right)}^{\frac{d}{4}}\theta \left(𝒳\right)⩽\left(K_{n}-1\right){\left(\frac{81\sigma_{n}^{2}}{\pi }\right)}^{\frac{d}{4}}M_{1}.$$

Similar to the proof of Lemma 2 in our paper,

$$\begin{array}{l} {ℛ}_{\phi }\left(\delta ,V_{\sigma_{n}}g\right)-{ℛ}_{\phi }(\delta ,f^{\delta })\\ ⩽\;\sum_{k=1}^{K_{n}}\;\;E\left[I[X\in {𝒳}_{k}^{\delta }]\sum_{j\neq k}^{K_{n}}\;\;|E[R|A\in G_{j}^{\delta },X]-E[R|A\in G_{k}^{\delta },X]|\cdot |\rangle W_{j},V_{\sigma_{n}}g(X)-f^{\delta }(X)\rangle |\right]\\ =\;\sum_{k=1}^{K_{n}}\;\;E\left[I[X\in {𝒳}_{k}^{\delta }]\sum_{j\neq k}^{K_{n}}\;\;|\eta_{j,k}^{\delta }(X)|\cdot |\left\langle W_{j},V_{\sigma_{n}}g(X)-f^{\delta }(X)\right\rangle |\right]. \end{array}$$

Now for $x\in {𝒳}_{k}^{\delta }$, we would bound $\left|\left\langle W_{j},V_{\sigma_{n}}g(x)-f^{\delta }(x)\right\rangle \right|$ when j≠k. We observe

$$\begin{array}{l} -1⩽\langle W_{j},V_{\sigma_{n}}g(x)\rangle \\ =\langle W_{j},{\left(\frac{2\sigma_{n}^{2}}{\pi }\right)}^{d/2}\int_{{\mathbb{R}}^{d}}e^{-2\sigma_{n}^{2}\|x-y{\|}^{2}}{\overset{\prime }{f}}^{\delta }(y)dy\rangle \\ ={\left(\frac{2\sigma_{n}^{2}}{\pi }\right)}^{d/2}\int_{{\mathbb{R}}^{d}}e^{-2\sigma_{n}^{2}\|x-y{\|}^{2}}\langle W_{j},{\overset{\prime }{f}}^{\delta }(y)\rangle dy\\ ={\left(\frac{2\sigma_{n}^{2}}{\pi }\right)}^{d/2}\int_{{\mathbb{R}}^{d}}e^{-2\sigma_{n}^{2}\|x-y{\|}^{2}}\langle W_{j},{\overset{\prime }{f}}^{\delta }(y)-\left(K_{n}-1\right)W_{j}\rangle dy+\left(K_{n}-1\right)\\ ⩽{\left(\frac{2\sigma_{n}^{2}}{\pi }\right)}^{d/2}\int_{B\left(x,\Delta^{\delta }(x)\right)}e^{-2\sigma_{n}^{2}\|x-y{\|}^{2}}\langle W_{j},{\overset{\prime }{f}}^{\delta }(y)-\left(K_{n}-1\right)W_{j}\rangle dy+\left(K_{n}-1\right)\\ ={\left(\frac{2\sigma_{n}^{2}}{\pi }\right)}^{d/2}\int_{B\left(x,\Delta^{\delta }(x)\right)}-K_{n}e^{-2\sigma_{n}^{2}\|x-y{\|}^{2}}dy+\left(K_{n}-1\right)\\ =-1+K_{n}{\mathbb{P}}_{\gamma \sigma_{n}}\left(\left|u|⩾\Delta^{\delta }(x\right)\right), \end{array}$$

where $\gamma_{\sigma_{n}}={\left(2\sigma_{n}^{2}/\pi \right)}^{d/2}e^{-2\sigma_{n}^{2}|u{|}^{2}}du$ is a spherical Gaussian in $R^{d}$. According to Ledoux and Talagrand (2013), we have $P_{\gamma_{\sigma_{n}}}(\left|u|⩾\Delta^{\delta }(x\right))⩽4e^{-\sigma_{n}^{2}\Delta^{\delta }(x{)}^{2}/2d}$ and consequently,

$$-1⩽\left\langle W_{j},V_{\sigma_{n}}g(x)\right\rangle ⩽-1+4K_{n}e^{-\sigma_{n}^{2}\Delta^{\delta }(x{)}^{2}/2d}.$$

Note that $\langle W_{j},f^{\delta }(x)\rangle =-1$ for $x\in {𝒳}_{k}^{\delta }$ and j≠k. Observe that above derivation holds for all $k\in \left[K_{n}\right]$. Hence, we conclude

$$\left|\left\langle W_{j},V_{\sigma_{n}}g(x)-f(x)\right\rangle \right|⩽4K_{n}e^{-\sigma_{n}^{2}\Delta_{k}^{\delta }(x{)}^{2}/2d}$$

for all $x\in {⋃}_{k=1}^{K_{n}}\;{𝒳}_{k}^{\delta }$. Then generalized geometric noise assumption for $t=\frac{2d}{\sigma_{n}^{2}}$ yields

$${ℛ}_{\phi }\left(\delta ,V_{\sigma_{n}}g\right)-{ℛ}_{\phi }(\delta ,f^{\delta })⩽4K_{n}(K_{n}-1)U(2d{)}^{qd/2}\sigma_{n}^{-qd}.$$

Therefore, combining both part, we conclude that

$${𝒜}_{\sigma_{n}}^{\delta }\left(\lambda_{n}\right)⩽cK_{n}^{2}\lambda_{n}^{q/(q+1)},$$

where c depends on the dimension of covariates d, geometric noise component q and associated constant U, and upper bound of volume of covariate space $M_{1}$ when we set $\sigma_{n}=\lambda_{n}^{-1/(q+1)d}$.

### Proof of Lemma 10:

To begin with, according to 1, it suffices to prove the result for ${\tilde{ℛ}}_{\phi }(\delta^{*},{\overset{ˆ}{f}}_{n}^{\delta^{*}})-{\tilde{ℛ}}_{\phi }^{*}$. Then, similar to the proof idea in Theorem 3.4 in Zhao et al. (2012), we observe the following decomposition:

$$\begin{array}{l} {\tilde{ℛ}}_{\phi }(\delta^{*},{\overset{ˆ}{f}}_{n}^{\delta^{*}})-{\tilde{ℛ}}_{\phi }^{*}⩽\left[\lambda_{n}{∥{\overset{ˆ}{f}}_{n}^{\delta^{*}}∥}_{{ℱ}_{n}}^{2}+{\tilde{ℛ}}_{\phi }(\delta^{*},{\overset{ˆ}{f}}_{n}^{\delta^{*}})-\underset{f\in {⊗}_{k=1}^{K_{n}-1}{ℋ}_{\kappa }^{k}}{inf}\;(\lambda_{n}∥f{∥}_{{ℱ}_{n}}^{2}+{\tilde{ℛ}}_{\phi }(\delta^{*},f))\right]\\ \;+\left[\underset{f\in {⊗}_{k=1}^{K_{n}-1}{ℋ}_{\kappa }^{k}}{inf}\;(\lambda_{n}∥f{∥}_{{ℱ}_{n}}^{2}+{\tilde{ℛ}}_{\phi }(\delta^{*},f))-{\tilde{ℛ}}_{\phi }^{*}\right], \end{array}$$

where the first term is referred as stochastic error and the second term is called the approximation bias term. We will bound each term separately in the following. First, the approximation bias term has been bounded by $𝒪(K_{n}^{2}\lambda_{n}^{q/(q+1)})$ in Lemma 9.

Next, to bound the stochastic error term, we follow the proof of Theorem 3.4 in Zhao et al. (2012). The proof of their theorem is basically derived by verifying the conditions of Theorem 5.6 in Steinwart and Scovel (2007). To achieve that, we first point out that, with Cauchy-Schwarz inequality, RAMSVM loss $(1-\gamma )\sum_{k\neq \delta^{*}(a)}\;{\left(1+\langle W_{j},f(x)\rangle \right)}^{+}+\gamma (K_{n}-{1-\langle W_{\delta^{*}(a)},f(x)\rangle )}^{+}$ is Lipschitz continuous with respect to f with Lipschitz constant $Z_{n}$. In addition, let $P^{\delta^{*}}$ be the population measure of $(X,\delta^{*}(a),R/p(\delta^{*}(a)|X)($ and $P_{n}^{\delta^{*}}$ be the associated empirical measure. Next, by the definition of ${\hat{f}}_{n}^{\delta^{*}}$, we have

$${\mathbb{P}}_{n}^{\delta^{\text{*}}}\left(\frac{R}{p\left(\delta^{\text{*}}\left(A\right)|X\right)}L_{\phi }\left(\delta^{\text{*}},{\hat{f}}_{n}^{\delta^{\text{*}}}\right)\right)+\lambda_{n}{\left\|{\hat{f}}_{n}^{\delta^{\text{*}}}\right\|}_{{ℱ}_{n}}^{2}⩽{\mathbb{P}}_{n}^{\delta^{\text{*}}}\left(\frac{R}{p\left(\delta^{\text{*}}\left(A\right)|X\right)}L_{\phi }\left(\delta^{\text{*}},f\right)\right)+\lambda_{n}{\left\|f\right\|}_{{ℱ}_{n}}^{2},$$

holds for any $f\in {⊗}_{k=1}^{K_{n}-1}{ℋ}_{\kappa }^{k}$. Hence, we can select f=0 to get

$${∥{\hat{f}}_{n}^{\delta^{*}}∥}_{{ℱ}_{n}}^{2}⩽\frac{1}{\lambda_{n}}\frac{1}{n}\sum_{i=1}^{n}\;\frac{R_{i}}{p(\delta^{*}(A_{i})|X_{i})}L_{\phi }(\delta^{*},0)⩽\frac{2}{\lambda_{n}}(K_{n}-1)Z_{n}.$$

Therefore, it suffices to consider a ball with radius $r=\sqrt{2\left(K_{n}-1\right)Z_{n}/\lambda_{n}}$ in the product $RKHS{⊗}_{k=1}^{K_{n}-1}{ℋ}_{\kappa }^{k}$, denoted by $B_{{⊗}_{k=1}^{K_{n}-1}{ℋ}_{\kappa }^{k}}(r)$. We define the function class

$${𝒢}_{\lambda_{n}}=\left\{\frac{R}{p\left(\delta^{\text{*}}\left(A\right)|X\right)}L_{\phi }\left(\delta^{\text{*}},f\right)+\lambda_{n}∥f{∥}_{{ℱ}_{n}}^{2}-\frac{R}{p\left(\delta^{\text{*}}\left(A\right)|X\right)}L_{\phi }\left(\delta^{\text{*}},f_{\lambda_{n}}^{\text{*}}\right)-\lambda_{n}{\left\|f_{\lambda_{n}}^{\text{*}}\right\|}_{{ℱ}_{n}}^{2}:f\in B_{{⊗}_{k=1}^{K_{n}-1}{ℋ}_{\kappa }^{k}}\left(r\right)\right\},$$

where $f_{\lambda_{n}}^{*}=arg{min}_{f}\;\left\{{ℛ}_{\phi }(\delta^{*},f)+\lambda_{n}∥f{∥}_{{ℱ}_{n}}^{2}:f\in B_{{⊗}_{k=1}^{Kn-1}{ℋ}_{\kappa }^{k}}(r)\right\}$. Then similar to the proof of Theorem 3.4 in Zhao et al. (2012), we verify the following three conditions: (i) By the Lipschitz continuity of $L_{\phi }$, there exists constant B such that ${sup}_{g\in {𝒢}_{\lambda_{n}}}\;∥g{∥}_{\infty }⩽B$ where $B=𝒪(\lambda_{n}^{-1/2})$; (ii) Based on the convexity of $L_{\phi }$, there exists constant c such that $E\left[g^{2}\right]⩽cE[g]$ for all $g\in {𝒢}_{\lambda_{n}}$ where $c=𝒪\left(\lambda^{-1}\right)$; (iii) Theorem 2.1 in Steinwart and Scovel (2007) gives that, for all $\sigma_{n}>0,0<v<2,\theta >0,\epsilon >0,{sup}_{P_{n}^{\delta^{*}}}\;log𝒩(B^{-1}{𝒢}_{\lambda_{n}},\epsilon ,L_{2}(P_{n}^{\delta^{*}}))⩽c_{2}\sigma_{n}^{(1-v/2)(1+\theta )d}\epsilon^{-v}$. Then the result follows from Theorem 5.6 in Steinwart and Scovel (2007).

Combining the bound for stochastic error term and approximation bias term, we complete the proof.

### Proof of Equation (10):

We firstly derive the equivalence of the two optimization problems based on the 0–1 loss. For any fixed δ,

$$\begin{array}{l} \;\underset{D_{g}}{min}\;E\left[\frac{(R\;-\;s(X){)}^{+}}{p(\delta (A)|X)}I\left[D_{g}(X)\neq \delta (A)\right]\right]+\left(K_{n}-1\right)E\left[\frac{(R\;-\;s(X){)}^{-}}{p(\delta (A)|X)}I[D_{g}(X)\neq \tilde{\delta }(A)]\right]\\ \;=\underset{D_{g}}{min}\;E_{\delta (A),X}\left[\frac{E\left[(R\;-\;s(X){)}^{+}|\delta (A),X\right]}{p(\delta (A)|X)}I\left[D_{g}(X)\neq \delta (A)\right]\right]\\ \;+\left(K_{n}-1\right)E_{\delta (A),X}\left[\frac{E\left[(R\;-\;s(X){)}^{-}|\delta (A),X\right]}{p(\delta (A)|X)}\sum_{j\neq \delta (A)}\;\;\frac{1}{K_{n}-1}I\left[D_{g}(X)\neq j\right]\right]\\ \;=\underset{D_{g}}{min}\;E_{\delta (A),X}\left[\frac{E\left[(R\;-\;s(X){)}^{+}|\delta (A),X\right]}{p(\delta (A)|X)}I\left[D_{g}(X)\neq \delta (A)\right]\right]\\ \;+E_{\delta (A),X}\left[\frac{E\left[(R\;-\;s(X){)}^{-}|\delta (A),X\right]}{p(\delta (A)|X)}\sum_{j\neq \delta (A)}\;\;I\left[D_{g}(X)\neq j\right]\right]\\ \;=\underset{D_{g}}{min}\;E_{X}\left[\sum_{i=1}^{K_{n}}\;\;E\left[(R\;-\;s(X){)}^{+}|\delta (A)=i,X\right]I\left[D_{g}(X)\neq i\right]\right]\\ \;+E_{X}\left[\sum_{i=1}^{K_{n}}\;\;E\left[(R\;-\;s(X){)}^{-}|\delta (A)=i,X\right]\sum_{j\neq i}\;\;I\left[D_{g}(X)\neq j\right]\right]\\ \;=\underset{D_{g}}{min}\;E_{X}\left[\sum_{i=1}^{K_{n}}\;\;E\left[(R\;-\;s(X){)}^{+}|\delta (A)=i,X\right]I\left[D_{g}(X)\neq i\right]\right]\\ \;+E_{X}\left[\sum_{i=1}^{K_{n}}\;\;E\left[(R\;-\;s(X){)}^{-}|\delta (A)=i,X\right](\sum_{j=1}^{K_{n}}\;\;I\left[D_{g}(X)\neq j\right]-I\left[D_{g}(X)\neq i\right])\right]\\ \;=\underset{D_{g}}{min}\;E_{X}\left[\sum_{i=1}^{K_{n}}\;\;E\left[(R\;-\;s(X){)}^{+}|\delta (A)=i,X\right]I\left[D_{g}(X)\neq i\right]\right]\\ \;+E_{X}\left[\sum_{i=1}^{K_{n}}\;\;E((R\;-\;s(X){)}^{-}|\delta (A)=i,X]\left(K_{n}-1-I\left[D_{g}(X)\neq i\right]\right)\right]\\ \;=\underset{D_{g}}{min}\;E_{X}\left[\sum_{i=1}^{K_{n}}\;\;E[R\;-\;s(X)|\delta (A)=i,X]I\left[D_{g}(X)\neq i\right]\right]\\ \;+\left(K_{n}-1\right)E_{X}\left[\sum_{i=1}^{K_{n}}\;\;E\left[(R\;-\;s(X){)}^{-}|\delta (A)=i,X\right]\right]\\ \;=\underset{D_{g}}{min}\;E\left[\frac{R\;-\;s(X)}{p(\delta (A)|X)}I\left[D_{g}(X)\neq \delta (A)\right]\right]\\ \;+\left(K_{n}-1\right)E_{X}\left[\sum_{i=1}^{K_{n}}\;\;E\left[(R\;-\;s(X){)}^{-}|\delta (A)=i,X\right]\right] \end{array}$$

Note that the second term in the last equation is not related to $D_{g}$. Hence, the problem is equivalent with minimizing the first term. This finishes the proof for the equivalence based on the 0–1 loss. The equivalence based on the RAMSVM loss can be directly guaranteed by Fisher consistency from Theorem 4.

### Derivations from Problem (9) to Dual Problems (11) and (12):

For the linear kernel, we solve (9) with its dual form. After introducing new slack variables ${\left(\xi_{i,j}\right)}_{i\in [n];j\in \left[K_{n}\right]}$, the problem with linear learning can be written as

$$\begin{array}{l} \;\underset{\beta_{k},\xi_{i,j}}{min}\;\frac{n\lambda }{2}\sum_{k=1}^{K_{n}-1}\;\;\beta_{k}^{T}\beta_{k}+\sum_{i=1}^{n}\;\;\omega_{i}\left[\gamma \xi_{i,\delta \left(A_{i}\right)}+(1-\gamma )\sum_{j\neq \delta \left(A_{i}\right)}\;\;\xi_{i,j}\right],\\ \;\text{s.t.}\;\xi_{i,j}⩾0\left(i\in [n],j\in \left[K_{n}\right]\right);\\ \xi_{i,\delta \left(A_{i}\right)}+\left\langle f\left(X_{i}\right),W_{\delta \left(A_{i}\right)}\right\rangle -\left(K_{n}-1\right)⩾0(i\in [n]);\\ \xi_{i,j}-\left\langle f\left(X_{i}\right),W_{j}\right\rangle -1⩾0\left(i\in [n],j\neq \delta \left(A_{i}\right)\right). \end{array}$$

The corresponding Lagrangian function $L_{a}$ is defined as

$$\begin{array}{l} L_{a}=\frac{n\lambda }{2}\sum_{k=1}^{K_{n}-1}\;\;\beta_{k}^{T}\beta_{k}+\sum_{i=1}^{n}\;\;\omega_{i}\left[\gamma \xi_{i,\delta \left(A_{i}\right)}+(1-\gamma )\sum_{j\neq \delta \left(A_{i}\right)}\;\;\xi_{i,j}\right]\\ \;-\sum_{i=1}^{n}\;\;\sum_{j=1}^{K_{n}}\;\;\rho_{i,j}\xi_{i,j}-\sum_{i=1}^{n}\;\;\alpha_{i,\delta \left(A_{i}\right)}\left[\xi_{i,\delta \left(A_{i}\right)}+\left\langle f\left(X_{i}\right),W_{\delta \left(A_{i}\right)}\right\rangle -\left(K_{n}-1\right)\right]\\ \;-\sum_{i=1}^{n}\;\;\sum_{j\neq \delta \left(A_{i}\right)}\;\;\alpha_{i,j}\left[\xi_{i,j}-\left\langle f\left(X_{i}\right),W_{j}\right\rangle -1\right], \end{array}$$

where ${\left(\alpha_{i,j}\right)}_{i\in [n];j\in \left[K_{n}\right]}$ and ${\left(\rho_{i,j}\right)}_{i\in [n];j\in \left[K_{n}\right]}$ are the Lagrangian multipliers. Furthermore, we can rewrite $L_{a}$ with

$$\begin{array}{l} L_{a}=\frac{n\lambda }{2}\sum_{k=1}^{K_{n}-1}\;\;\beta_{k}^{T}\beta_{k}+\sum_{i=1}^{n}\;\;\sum_{j=1}^{K_{n}}\;\;\left[\omega_{i}\left(\gamma I\left[j=\delta \left(A_{i}\right)\right]+(1-\gamma )I\left[j\neq \delta \left(A_{i}\right)\right]\right)-\rho_{i,j}-\alpha_{i,j}\right]\xi_{i,j}\\ \;+\sum_{i=1}^{n}\;\;\alpha_{i,\delta \left(A_{i}\right)}\left(K_{n}-1\right)+\sum_{i=1}^{n}\;\;\sum_{j\neq \delta \left(A_{i}\right)}\;\;\alpha_{i,j}\\ \;-\sum_{i=1}^{n}\;\;\alpha_{i,\delta \left(A_{i}\right)}\left\langle f\left(X_{i}\right),W_{\delta \left(A_{i}\right)}\right\rangle +\sum_{i=1}^{n}\;\;\sum_{j\neq \delta \left(A_{i}\right)}\;\;\alpha_{i,j}\left\langle f\left(X_{i}\right),W_{j}\right\rangle . \end{array}$$

Next we take partial derivative of $L_{a}$ with respect to ${\left(\xi_{i,j}\right)}_{i\in [n];j\in \left[K_{n}\right]}$ and ${\left(\beta_{k}\right)}_{k\in \left[K_{n}-1\right]}$, and let them be 0. We have

$$\frac{\partial L_{a}}{\partial \xi_{i,j}}=\omega_{i}\left(\gamma I\left[j=\delta \left(A_{i}\right)\right]+(1-\gamma )I\left[j\neq \delta \left(A_{i}\right)\right]\right)-\rho_{i,j}-\alpha_{i,j}=0\left(i\in [n],j\in \left[K_{n}\right]\right),$$

and

$$\frac{\partial L_{a}}{\partial \beta_{k}}=n\lambda \beta_{k}-\sum_{i=1}^{n}\;\alpha_{i,\delta \left(A_{i}\right)}W_{\delta \left(A_{i}\right),k}X_{i}+\sum_{i=1}^{n}\;\sum_{j\neq \delta \left(A_{i}\right)}\;\alpha_{i,j}W_{j,k}X_{i}=0\left(k\in \left[K_{n}-1\right]\right).$$

When the partial derivatives for ${\left(\beta_{k}\right)}_{k\in \left[K_{n}-1\right]}$ equal to 0, we have

$$\beta_{k}=\frac{1}{n\lambda }\left[\sum_{i=1}^{n}\;\;\alpha_{i,\delta \left(A_{i}\right)}W_{\delta \left(A_{i}\right),k}X_{i}-\sum_{i=1}^{n}\;\;\sum_{j\neq \delta \left(A_{i}\right)}\;\;\alpha_{i,j}W_{j,k}X_{i}\right].$$

After plugging the above characterizations of β into $L_{a}$, we can simplify $L_{a}$ with

$$L_{a}=-\frac{n\lambda }{2}\sum_{k=1}^{K_{n}-1}\;\beta_{k}^{T}\beta_{k}+\sum_{i=1}^{n}\;\alpha_{i,\delta \left(A_{i}\right)}\left(K_{n}-1\right)+\sum_{i=1}^{n}\;\sum_{j\neq \delta \left(A_{i}\right)}\;\alpha_{i,j}.$$

Note that maximizing $L_{a}$ with respect to $\alpha_{i,j}$ is equivalent to minimizing $-L_{a}$, which solves the dual problem (11). The constraints of problem (11) come from ${\left(\alpha_{i,j}\right)}_{i\in [n];j\in \left[K_{n}\right]}⩾0$, ${\left(\rho_{i,j}\right)}_{i\in [n];j\in \left[K_{n}\right]}⩾0$, and ${\left(\partial L_{a}/\partial \xi_{i,j}\right)}_{i\in [n];j\in \left[K_{n}\right]}=0$.

For the general kernel, we similarly introduce the slack variables ${\left(\xi_{i,j}\right)}_{i\in [n];j\in \left[K_{n}\right]}$. If the intercepts ${\left(\theta_{k,0}\right)}_{k\in \left[K_{n}-1\right]}$ are included in the penalty term, then (9) is equivalent to

$$\begin{array}{l} \;\underset{\theta_{k},\theta_{k,0},\xi_{i,j}}{min}\;\frac{n\lambda }{2}\sum_{k=1}^{K_{n}-1}\;\;\theta_{k}^{T}G\theta_{k}+\frac{n\lambda }{2}\sum_{k=1}^{K_{n}-1}\;\;\theta_{k,0}^{2}+\sum_{i=1}^{n}\;\;\omega_{i}\left[\gamma \xi_{i,\delta \left(A_{i}\right)}+(1-\gamma )\sum_{j\neq \delta \left(A_{i}\right)}\;\;\xi_{i,j}\right],\\ \;\text{s.t.}\;\xi_{i,j}⩾0\left(i\in [n],j\in \left[K_{n}\right]\right);\\ \xi_{i,\delta \left(A_{i}\right)}+\left\langle f\left(X_{i}\right),W_{\delta \left(A_{i}\right)}\right\rangle -\left(K_{n}-1\right)⩾0(i\in [n]);\\ \xi_{i,j}-\left\langle f\left(X_{i}\right),W_{j}\right\rangle -1⩾0\left(i\in [n],j\neq \delta \left(A_{i}\right)\right). \end{array}$$

Similar to the linear case, we introduce the Lagrangian multipliers ${\left(\rho_{i,j}\right)}_{i\in [n];j\in \left[K_{n}\right]}$ and ${\left(\alpha_{i,j}\right)}_{i\in [n];j\in \left[K_{n}\right]}$, calculate partial derivative with respect to ${\left(\theta_{q}\right)}_{q\in \left[K_{n}-1\right]},{\left(\theta_{q,0}\right)}_{q\in \left[K_{n}-1\right]}$, and ${\left(\xi_{i,j}\right)}_{i\in [n];j\in \left[K_{n}\right]}$, and set the derivatives to 0. Then we have that

$$\theta_{k}=\frac{1}{n\lambda }G^{-1}\left[\sum_{i=1}^{n}\;\;\alpha_{i,\delta \left(A_{i}\right)}W_{\delta \left(A_{i}\right),k}G_{i}-\sum_{i=1}^{n}\;\;\sum_{j\neq \delta \left(A_{i}\right)}\;\;\alpha_{i,j}W_{j,k}G_{i}\right],$$

and

$$\theta_{k,0}=\frac{1}{n\lambda }\left[\sum_{i=1}^{n}\;\;\alpha_{i,\delta \left(A_{i}\right)}W_{\delta \left(A_{i}\right),k}-\sum_{i=1}^{n}\;\;\sum_{j\neq \delta \left(A_{i}\right)}\;\;\alpha_{i,j}W_{j,k}\right],$$

where $G_{i}$ is the i-th column of G. After plugging the above characterizations of θ into the Lagrangian function and rewriting it, we get (12).

## Appendix D. Additional Simulation Results

### D.2. Simulations Under More Unbalanced Designs.

To better demonstrate the performance of GROWL, we conduct several other simulations under more unbalanced designs for Scenario 3 in homogeneous setting. For the unbalanced design (II), the propensity scores of 15 treatments are set to be ((0.035, 0.035, 0.035, 0.114, 0.114), (0.035, 0.035, 0.035, 0.114, 0.114), (0.035, 0.035, 0.088, 0.088, 0.088)), and for the unbalanced design (III), the propensity scores are set to be ((0.020, 0.020, 0.020, 0.137, 0.137), (0.020, 0.020, 0.020, 0.137, 0.137), (0.020, 0.020, 0.098, 0.098, 0.098)). Varying from balanced design, unbalanced design (I) (same setting as shown in Section 4.1), unbalanced design (II), and unbalanced design (III), the treatment propensities become more and more unbalanced. In addition, we conduct another simulation setting where the propensity scores of one of the three treatment groups is extremely small. Specifically, for this extreme case, the propensity scores are ((0.070, 0.070, 0.087, 0.087, 0.087), (0.100, 0.100, 0.100, 0.100, 0.100),(0.020, 0.020, 0.020, 0.020, 0.020)). Overall, GROWL still performs the best in most cases shown in Figures 6 and 7. As treatment propensities become more unbalanced, GROWL may need more training data in order to learn the true partition $\delta^{0}$ correctly. In addition, a roughly correct ${\hat{\delta }}_{n}$ is still helpful in terms of the performance of the value function. Compared with other methods that do not consider the treatment structure, GROWL is able to combine the similar treatments into the treatment groups. The decision rules learned from GROWL perform better because they are estimated under the treatment groups that have more observations than the individual treatments.

### D.3. Simulations When Propensity Scores Depend on the Covariates.

We conduct more general simulation settings when the treatment propensity scores depend on the covariates. Specifically, the treatment propensity score p(a∣x) is provided with the following multinomial model:

$$\log\frac{p\left(a|X\right)}{p\left(1|X\right)}=\beta_{a,0}+\beta_{a,1}X_{1}+\beta_{a,2}X_{2}+\cdots +\beta_{a,10}X_{10},$$

for a=2,…,M, where $\beta_{i,j}’$ s are all generated independently from U[-0.1,0.1]. For the homogeneous case, we take Scenario 1 as an example. The value shown in Figure 8 demonstrates that GROWL still has superior performance over other methods. For the non-homogeneous case in Scenario 4, as shown in Figure 9, the overall trend of GROWL is similar to that of the unbalanced design in Figure 2. GROWL is still competitive, and especially has smaller variance than other methods.

### D.4. Estimated Coefficients in STAR*D Analysis.

**Table 3:** Estimated coefficients of linear comparison function for GROWL on the STAR*D dataset. Larger coefficients encourage better reward.

Variable Name	Group 1	Group 2	Group 3	Group 4	Group 5

intercept	0.713	0.168	0.658	0.191	−1.730
gender (female)	−0.839	0.249	1.635	−0.444	−0.601
ethnic (white)	−0.878	−0.180	−0.764	0.221	1.601
age	−0.959	−0.319	0.579	−0.746	1.445
depression history (yes)	−1.427	0.021	−0.368	0.528	1.245
marital	1.049	0.371	−1.636	0.282	−0.066
school years	1.421	−0.151	−0.033	0.153	−1.390
education	0.526	−1.230	1.166	−0.841	0.379
student (yes)	−0.813	1.704	−0.541	−0.377	0.026
employment	0.178	−0.867	−1.085	1.369	0.405
volunteer work	0.066	−0.750	−0.056	−0.886	1.626
QIDS change during Level 1	−1.574	0.136	1.206	0.228	0.004
QIDS at the start of Level 2	0.743	0.576	−0.952	0.851	−1.218

## Appendix E. Implementation Details

**Algorithm 1:** GROWL using the Genetic Algorithm

Initialize.
a. Fit (penalized) linear regression R~X with training data and get residuals r;
b. Input the estimated initial partition set $\Delta_{0}$.
For each partition δ in $\Delta_{0}$, **do**
a. Fit treatment A into group δ(A) based on δ
b. **If** r>0, Stay with the same assigned treatment δ(A), and set $\overset{‾}{\delta }(A)=\delta (A)$;
**Else** Uniformly switch δ(A) to arbitrary unassigned treatment $\tilde{\delta }(A)$, and set $\overset{‾}{\delta }(A)=\tilde{\delta }(A)$;
c. Use $\left(R,\overset{‾}{\delta }(A),X\right)$ to fit RAMSVM with weights $w_{G}=\frac{\left|r\right|}{p(\delta (A)|X)};$
d. Get fitted decision function ${\overset{ˆ}{f}}^{\delta }$ based on RAMSVM;
e. Plug $\left(\delta ,{\overset{ˆ}{f}}^{\delta }\right)$ back into the empirical average of the risk function ${\tilde{ℛ}}_{\phi }$;
f. Get the risk value for δ.
**Updated** the set Δ using the **Genetic Algorithm** until convergence;
**Obtain** the optimal $\hat{\delta }$ and corresponding ${\hat{D}}_{g}$ under group domain;
**Sample** one treatment from group ${\hat{D}}_{g}(X)$ based on $\pi_{\delta }$, and finally get the ITR D(X).

**Algorithm 2:** GROWL with the Greedy Adjustment

Initialize.
a. Fit (penalized) linear regression R~X with training data and get residuals r;
b. Input the estimated initial partition set $\Delta_{0}$.
**Update** δ, **do**
**For** $j=1,\;2,\ldots ,M_{n},1,\;2,\ldots ,M_{n},\ldots$, **do**
a. Adjust the assignment of the *j*-th treatment and hold the assignment for others fixed;
b. Get the risk value for the adjusted δ in the same way shown in Algorithm 1;
c. Obtain the locally best δ in cyclic fashion.
**Until** convergence.
**Obtain** the optimal $\overset{ˆ}{\delta }$ and corresponding ${\overset{ˆ}{D}}_{g}$ under group domain;
**Sample** one treatment from group ${\hat{D}}_{g}(X)$ based on $\pi_{\delta }$, and finally get the ITR D(X).
